# Prognostic and therapeutic role of targetable lesions in B-lineage acute lymphoblastic leukemia without recurrent fusion genes

**DOI:** 10.18632/oncotarget.7356

**Published:** 2016-02-12

**Authors:** Monica Messina, Sabina Chiaretti, Jiguang Wang, Anna Lucia Fedullo, Nadia Peragine, Valentina Gianfelici, Alfonso Piciocchi, Fulvia Brugnoletti, Filomena Di Giacomo, Simona Pauselli, Antony B. Holmes, Maria Cristina Puzzolo, Giulia Ceglie, Valerio Apicella, Marco Mancini, Geertruy te Kronnie, Anna Maria Testi, Antonella Vitale, Marco Vignetti, Anna Guarini, Raul Rabadan, Robin Foà

**Affiliations:** ^1^ Hematology, Department of Cellular Biotechnologies and Hematology, Sapienza University, Rome, Italy; ^2^ Department of Systems Biology, Biomedical Informatics and Center for Computational Biology and Bioinformatics, Columbia University, New York, NY, USA; ^3^ GIMEMA Data Center, Rome, Italy; ^4^ Department of Molecular Biotechnology and Health Science, and Center for Experimental Research and Medical Studies (CeRMS), University of Torino, Torino, Italy; ^5^ Institute for Cancer Genetics and The Herbert Irving Comprehensive Cancer Center, Columbia University, New York, NY, USA; ^6^ Department of Women's and Children's Health, University of Padova, Padova, Italy

**Keywords:** acute lymphoblastic leukemia, next generation sequencing, copy number aberrations, novel prognostic markers, genetic-driven targeted therapy

## Abstract

To shed light into the molecular bases of B-lineage acute lymphoblastic leukemia lacking known fusion transcripts, i.e. *BCR-ABL1*, *ETV6-RUNX1*, *E2A-PBX1*, and *MLL* rearrangements (B-NEG ALL) and the differences between children, adolescents/young adults (AYA) and adults, we analyzed 168 B-NEG ALLs by genome-wide technologies. This approach showed that B-NEG cases carry 10.5 mutations and 9.1 copy-number aberrations/sample. The most frequently mutated druggable pathways were those pertaining to RAS/RTK (26.8%) and JAK/STAT (12.5%) signaling. In particular, *FLT3* and JAK/STAT mutations were detected mainly in AYA and adults, while *KRAS* and *NRAS* mutations were more frequent in children. RAS/RTK mutations negatively affected the outcome of AYA and adults, but not that of children. Furthermore, adult B-NEG ALL carrying JAK/STAT mutations had a shorter survival. *In vitro* experiments showed that FLT3 inhibitors reduced significantly the proliferation of *FLT3*-mutated primary B-NEG ALL cells. Likewise, PI3K/mTOR inhibitors reduced the proliferation of primary cells harboring *RAS* and *IL7R* mutations. These results refine the genetic landscape of B-NEG ALL and suggest that the different distribution of lesions and their prognostic impact might sustain the diverse outcome between children, adults and partly AYA - whose genomic scenario is similar to adults - and open the way to targeted therapeutic strategies.

## INTRODUCTION

B-lineage acute lymphoblastic leukemia (B-ALL) represents 75-85% of all ALLs and is characterized by the accumulation of immature B cells, which results in the suppression of normal hematopoiesis. B-ALL is the most frequent cancer in children, with the highest incidence between 2 and 5 years, while it is relatively rare in adults, with a peak after the age of 50 [1-3]. While in childhood the survival rates are in the order of 80%, the outcome in adults is still unsatisfactory, with long-term survival rates of about 40-50% of cases [4-8]. An intermediate group is represented by the adolescents/young adults (AYA), who show an improved outcome when treated with pediatric-like protocols [9-11].

Besides the different prognosis, children and adults are characterized by a variable distribution of recurrent chromosomal aberrations with prognostic impact, such as high hyperdiploidy and *ETV6-RUNX1* transcript, common in children, *BCR-ABL1* and *MLL* rearrangements, more frequent in adults [3, 12-20]. While these lesions characterize about 50% of B-ALL, a considerable proportion of cases proves negative for gold-standard molecular screening (B-NEG ALL). The definition of the genetic landscape of this “genetically-orphan” subgroup has partially improved by the introduction of genome-wide technologies, such as gene expression profiling (GEP), single nucleotide polymorphism (SNP) arrays and next generation sequencing (NGS) [21-26]. The latter has clarified the molecular background of specific subgroups, such as “*BCR-ABL1*-like” and hypodiploid ALL, while a comprehensive description of B-NEG has so far not been provided [25-26].

We analyzed 168 B-NEG ALLs (50 children, 61 AYA, 57 adults) - distributed in a discovery panel (*N* = 13), screening panel 1 (*N* = 68) and screening panel 2 (*N* = 87) - by NGS and copy number aberration (CNA) analysis to refine the molecular scenario of B-NEG ALL cases and assess if different lesions might account for the different outcome between children, AYA and adults, with the ultimate goal of investigating the potential role of targeted therapeutic approaches.

## RESULTS

### Incidence and outcome of B-NEG ALLs stratified for age cohorts

**Figure 1:**
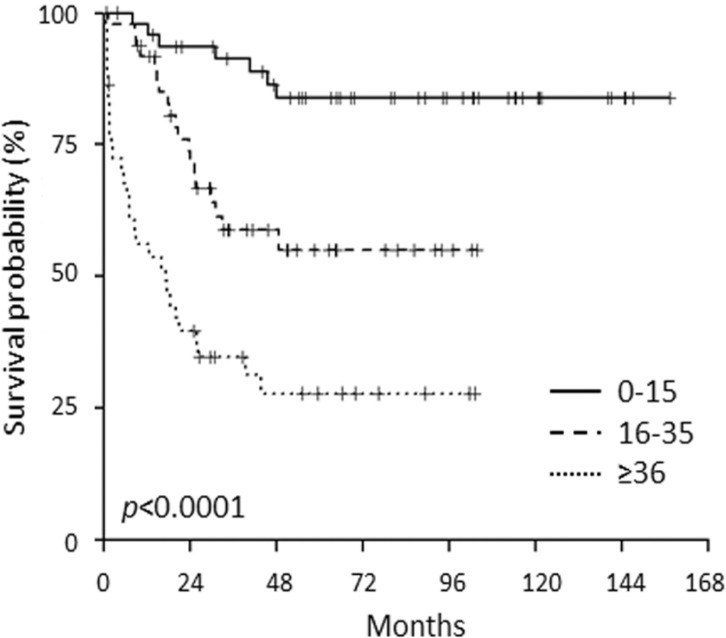
OS of B-NEG ALL patients, included in the present study, stratified for age cohorts

The meta-analysis of the incidence of known molecular aberrations in a cohort of over 5,000 ALL [3] revealed that within B-ALL, B-NEG cases represent 70.5% of childhood, 72.7% of AYA and 42.7% of adults. Furthermore, within the population included in the current study, we evaluated the outcome of the above mentioned age cohorts. Clinical outcome data were available for 142 patients (48 children, 50 AYA and 44 adults) with a median follow-up of 65 months. Overall survival (OS) was significantly different among children (83%), AYA (55%) and adults (29%), resembling that of ALL in general (Figure 1) [3].

### Genomic overview of B-NEG ALL

**Figure 2:**
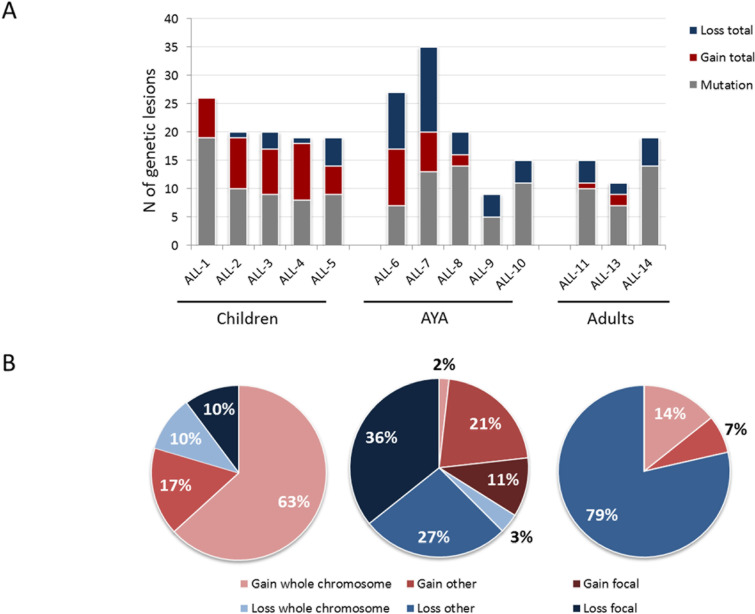
Overall load of genetic lesions of B-NEG ALL **A.** Load of somatic mutations and CNAs across different age cohorts. **B.** CNAs distribution and type across different age cohorts.

Whole exome sequencing (WES) of the discovery panel cases revealed 136 mutated genes (10.5/case), with a similar load across the 3 age cohorts (Figure 2A). Mutated genes were highly heterogeneous across B-NEG ALL samples with only *FLT3*, *PREX2, PAX5* - recurrently mutated, being found in 2 of the 13 samples (Table S1). In spite of this, *in silico* analyses revealed a significant enrichment of the following gene categories: focal adhesion/ECM interaction, small GTPase mediated signal transduction, ion transport and protein kinase activity, as described in Supplemental Material and Table S2.

**Figure 3:**
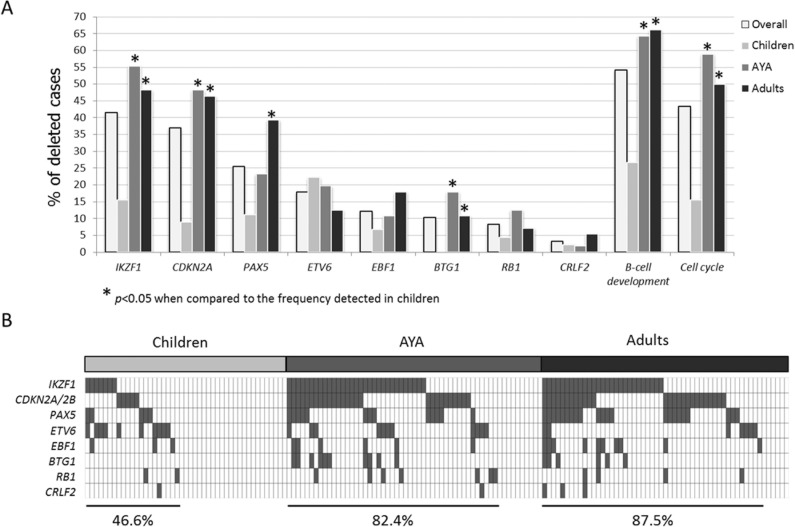
Incidence of the most common deletions in B-NEG ALL **A.** Frequency of deletions targeting *IKZF1*, *CDKN2A*, *PAX5*, *ETV6*, *EBF1*, *BTG1*, *RB1*, *CRLF2* - and grouped in functional categories - in the whole population and in age cohorts. Asterisks indicate deletions with a statistically significant different incidence when comparing children and AYA or children and adults. **B.** Distribution of the abovementioned deletions in children, AYA and adult populations.

By SNP arrays we detected 119 CNAs (9.1 CNAs/sample, range 4-22). The load and type of CNAs was heterogeneous across the age cohorts. Indeed, whilst childhood ALLs were affected by 9.8 CNAs, adult ALLs carried 4.6 CNAs on average. AYA displayed an intermediate behavior, with 2/5 samples being genetically complex and 3 harboring a number of CNAs similar to the adult cohort (Figure 2A). Pediatric samples were mostly characterized by gains of entire chromosomes. Contrariwise, AYA and adult patients were mainly affected by losses of variable size, ranging from deletions of chromosome arms to single exons (Figure 2B). The most common minimally deleted regions were those encompassing known target genes [21, 22]. Indeed, the screening of these known targets confirmed that the most frequently deleted genes were *IKZF1* (41.4%), *CDKN2A* (36.9%), *PAX5* (25.5%) and *ETV6* (17.8%) (Figure 3A).

### Identification and screening of the recurrently mutated genes

Given the low number of recurrently mutated genes detected in the discovery panel, WES was broadened to 68 additional B-NEG cases (screening panel 1) and the bioinformatic analysis focused on the genes mutated in the discovery panel and/or reported as affected by mutations or CNAs in published ALL series [25-29]. This approach yielded a shorter list of genes frequently targeted by mutations, which overall clustered in the RAS/RTK and JAK/STAT cascades, or affected B-lineage differentiation, and also highlighted intriguing differences amongst the age cohorts (Table S3).

To validate the latter findings we sequenced the most frequently mutated genes within the abovementioned pathways - namely *FLT3*, *KRAS*, *NRAS, IL7R*, *JAK2, CRLF2, JAK1* and *PAX5* - in 87 additional B-NEG ALLs (screening panel 2). The results described below refer to a total population of 168 cases (50 children, 61 AYA and 57 adults).

### Age-related genetic lesions in B-NEG ALL

#### Genetic lesions of PAX5

Among the mutations of B-lineage development genes, those affecting *PAX5* were the most common, occurring in 20 patients (11.9%). Mutations clustered in the paired-box domain with roughly 50% being represented by the hotspot P80R (Figure S1A). The comparison among age groups highlighted a significant difference between the percentage of mutations detected in children and adults (2% *vs* 19.3%, *p* = 0.005, Figure S1B); AYA displayed an intermediate behavior, with mutations observed in 13.1% of cases.

By integrating the mutational with the CN status, we confirmed that *PAX5* lesions increase with age progression, being detected in 13.3% of children, 28.6% of AYA and 44.6% of adults: the difference was statistically significant between children and adults, as well as by combining AYA and adults (*p* = 0.001, *p* = 0.004, Figure S1C).

#### Recurrent deletions of B-cell differentiation and cell-cycle genes

Beside *PAX5* deletions, the screening of CNAs of selected genes involved in B-cell development (i.e. *IKZF1*, *EBF1*, *BTG1*) as well as the cell-cycle related genes (i.e. *CDKN2A/B* and *RB1*) revealed that deletions were significantly more frequent in AYA and adult patients than in children. Overall, B-cell development genes were deleted in 26.6% of pediatric, 64.3% of AYA and 66.1% of adult cases. Similarly, 15.5% of pediatric, 58.9% of AYA and 50% of adult patients harbored deletions targeting cell-cycle genes. In particular, these differences achieved a statistical significance for *IKZF1*, *BTG1* and *CDKN2A*, as detailed in Figure 3A.

As a result, these selected CNAs tended to accumulate with age, with 46.6% of children, 82.4% of AYA and 87.5% carrying at least one CNA (Figure 3B).

#### Distribution and features of RAS/RTK pathway mutations

**Figure 4:**
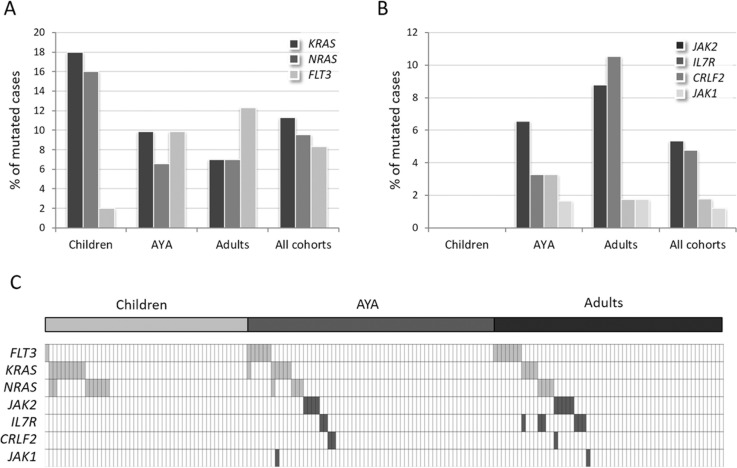
Incidence of RAS/RTK and JAK/STAT pathway mutations in B-NEG ALL **A.** Frequency of mutations targeting *KRAS*, *NRAS* and *FLT3*. **B.** Frequency of mutations targeting *JAK2*, *IL7R*, *CRLF2*, *JAK1*. **C.** Distribution of the mutations targeting the RAS/RTK pathway (light grey cells) and JAK/STAT signaling (dark grey cells).

Mutations of *FLT3*, *KRAS* and *NRAS*, grouped in the RAS/RTK pathway, were detected in 26.8% (45/168) of cases. In particular, *KRAS* was mutated in 11.3% (19/168) of cases, *NRAS* in 9.5% (16/168) and *FLT3* in 8.3% (14/168) (Figure 4A). The latter was prevalently mutated in adults (12.3%) and AYA (9.8%), while only 2% of children carried *FLT3* mutations (Figure 4A). This difference reached statistical significance when comparing adults *vs* children, as well as by combining AYA and adults (*p* = 0.046 and *p* = 0.043, Figure S2A). *FLT3* mutations mainly targeted the tyrosine kinase catalytic domain (TKD, 50%) or were represented by internal tandem duplications (ITD) in the juxtamembrane domain (36%) (Table 1, Figure S2B).

*KRAS* and *NRAS* mutations accumulated mostly in childhood: in fact, collectively *RAS* mutations were detected in 30% of children and in 14.7% of AYA and 14% of adults (*p* = 0.052, *p* = 0.045).

Mutations targeted almost exclusively (92%) the known hotspot G12 and G13, and in the remaining cases the aminoacidic residues 60-63 (Table 1, Figure S3).

Finally, we found no overlap between *FLT3* mutations and *NRAS*, *KRAS* G12-13 mutations; nonetheless, 1 sample carrying a *FLT3* ITD and *KRAS* G60D mutations was detected. Moreover, 3 cases harbored both *NRAS* and *KRAS* mutations. Notably, concomitant *NRAS* and *KRAS* mutations were only observed in AYA and children.

Of interest, cytogenetic analyses - successful in 109/168 cases - revealed a hyperdiploid karyotype in 17, with only 5 of them harboring RAS/RTK mutations. Given the known association between hyperdiploidy and RAS/RTK mutations [30-36], the analysis was repeated excluding hyperdiploid cases and samples lacking a cytogenetic evaluation, a similar incidence of RAS/RTK-mutated cases was observed: *KRAS/NRAS* mutations were detected in 32% of children, 15.6% of AYA and 15.3% of adults, while *FLT3* mutations were detected in 12.5% of AYA and 15.3% of adults.

**Table 1:** Features of the mutations affecting RAS/RTK pathway

ID	Age	Cohort	Panel	Gene	Exon	AA change	Type of mutation	Domain (Uniprot)
GQW_63	4	Children	Discovery	*FLT3*	20	M837-	In frame deletion	TKD
GQW_71	19	AYA	Discovery	*FLT3*	20	Y842N	Missense	TKD
GQW_220	16	AYA	Screening 1	*FLT3*	14	Y572C	Missense	JM
GQW_210	22	AYA	Screening 1	*FLT3*	14	ITD	ITD	JM
GQW_222	23	AYA	Screening 1	*FLT3*	14	ITD	ITD	JM
GQW_236	30	AYA	Screening 1	*FLT3*	16	N676K	Missense	TKD
ALL_132	20	AYA	Screening 2	*FLT3*	20	D835H	Missense	TKD
GQW_187	56	Adults	Screening 1	*FLT3*	14	ITD	ITD	JM
GQW_199	59	Adults	Screening 1	*FLT3*	20	D835Y	Missense	TKD
GQW_230	39	Adults	Screening 1	*FLT3*	11	S446L	Missense	Extracellular
ALL_14	78	Adults	Screening 2	*FLT3*	14	ITD	ITD	JM
ALL_19	40	Adults	Screening 2	*FLT3*	20	p.837_838delMSinsVQG	In frame indel	TKD
ALL_104	65	Adults	Screening 2	*FLT3*	14	ITD	ITD	JM
ALL_125	40	Adults	Screening 2	*FLT3*	21	P857S	Missense	TKD
GQW_249	12	Children	Screening 1	*KRAS*	2	G12D	Missense	GTPase
GQW_245	3	Children	Screening 1	*KRAS*	2	G13D	Missense	GTPase
GQW_248	9	Children	Screening 1	*KRAS*	2	G13D	Missense	GTPase
ALL_142	3	Children	Screening 2	*KRAS*	2	G13D	Missense	GTPase
WB_6_PD	3	Children	Screening 2	*KRAS*	2	G12D	Missense	GTPase
WB_8_PD	1	Children	Screening 2	*KRAS*	2	G12R	Missense	GTPase
WB_10_PD	3	Children	Screening 2	*KRAS*	2	G12S	Missense	GTPase
WB_11_PD	14	Children	Screening 2	*KRAS*	2	G12D	Missense	GTPase
WB_14_PD	5	Children	Screening 2	*KRAS*	2	G12D	Missense	GTPase
GQW_208	19	AYA	Screening 1	*KRAS*	3	E63K	Missense	GTPase
GQW_210	22	AYA	Screening 1	*KRAS*	3	G60D	Missense	GTPase
GQW_221	16	AYA	Screening 1	*KRAS*	2	G12D	Missense	GTPase
GQW_225	28	AYA	Screening 1	*KRAS*	2	G12D	Missense	GTPase
ALL_138	21	AYA	Screening 2	*KRAS*	2	G12V	Missense	GTPase
ALL_106	20	AYA	Screening 2	*KRAS*	2	G12D	Missense	GTPase
GQW_81	53	Adults	Discovery	*KRAS*	4	A146T	Missense	GTPase
GQW_196	58	Adults	Screening 1	*KRAS*	2	G12S	Missense	GTPase
ALL_91	48	Adults	Screening 2	*KRAS*	2	G13D	Missense	GTPase
ALL_120	47	Adults	Screening 2	*KRAS*	2	G12V	Missense	GTPase
GQW_247	2	Children	Screening 1	*NRAS*	2	G12A	Missense	GTPase
GQW_242	14	Children	Screening 1	*NRAS*	2	G13D	Missense	GTPase
GQW_254	9	Children	Screening 1	*NRAS*	2	G13D	Missense	GTPase
ALL_111	9	Children	Screening 2	*NRAS*	2	G13D	Missense	GTPase
ALL_146	14	Children	Screening 2	*NRAS*	3	Q61R	Missense	GTPase
WB_2_PD	11	Children	Screening 2	*NRAS*	2	G13D	Missense	GTPase
WB_6_PD	3	Children	Screening 2	*NRAS*	2	G12S	Missense	GTPase
WB_14_PD	5	Children	Screening 2	*NRAS*	2	G13D	Missense	GTPase
GQW_225	28	AYA	Screening 1	*NRAS*	2	G13D	Missense	GTPase
GQW_229	30	AYA	Screening 1	*NRAS*	2	G12D	Missense	GTPase
ALL_88	23	AYA	Screening 2	*NRAS*	2	G12D	Missense	GTPase
ALL_116	16	AYA	Screening 2	*NRAS*	2	G12D	Missense	GTPase
GQW_181	44	Adults	Screening 1	*NRAS*	2	G12A	Missense	GTPase
GQW_198	42	Adults	Screening 1	*NRAS*	2	G13D	Missense	GTPase
ALL_29	42	Adults	Screening 2	*NRAS*	2	G12S	Missense	GTPase
ALL_134	78	Adults	Screening 2	*NRAS*	2	G12D	Missense	GTPase

#### Distribution and features of JAK/STAT pathway mutations

Mutations in genes converging into the JAK/STAT pathway - *JAK2*, *IL7R*, *CRLF2* and *JAK1 -* accounted for 12.5% of cases (21/168). Missense mutations targeting arginine 863 of *JAK2* and indels in exon 6 of *IL7R* were the most frequent, detected in 5.3% and 4.8% of cases, respectively. Less than 2% of cases harbored *CRLF2* F232C mutations (*N* = 3) or *JAK1* mutations (*N* = 2) (Table 2). JAK/STAT mutations were uniquely detected in patients >15 years (*p* < 0.001): none was in fact found in children, whereas 13% of AYA and 17.5% of adult patients were affected (Figure 4B). Furthermore, JAK/STAT mutations were associated with male gender (*p* = 0.01) and higher white blood cell (WBC) counts (90.6 *vs* 43.5 ×10^9^/L in mutated *vs* WT cases, *p* = 0.002). GEP analysis of JAK/STAT-mutated samples, revealed that 10 of the 21 mutated samples (47.6%) displayed a *BCR-ABL1*-like profile, thus indicating that JAK/STAT mutations, though characterizing the *BCR-ABL1*-like cases, are not exclusive of this cohort (Figure S4).

Moreover, when analyzed in light of other features bearing a negative prognostic impact [23-25], 57.1% (12/21) carried an *IKZF1* deletion and 3 harbored also a *CRLF2-P2RY8* rearrangement (Figure S4). The latter aberration was overall detected in 5 (3 adults, 1 AYA and 1 child) out of 157 samples analyzed by MLPA.

Mutations targeting the JAK/STAT genes were mainly mutually exclusive, with only 1 sample carrying 2 mutations: one targeting *JAK2* and the other *CRLF2*, the latter detected at the subclonal level.

Moreover, only 4 cases displayed concomitant RAS/RTK and JAK/STAT pathway mutations: in 2 cases we found *IL7R* and *NRAS* mutations, in 1 sample *IL7R* and *KRAS* mutations, and, lastly, 1 case harbored *JAK1* and *KRAS* mutations (Figure 4C).

Contrariwise, *PAX5* mutations frequently overlapped with RAS/RTK (8/45) and JAK/STAT (7/21) mutations.

**Table 2:** Features of the mutations affecting JAK/STAT pathway

ID	Age	Cohort	Panel	Gene	Ex on	AA change	Type of mutation	Domain (Uniprot)
GQW_211	22	AYA	Screening 1	*JAK2*	16	I682F	Missense	TK1
GQW_239	19	AYA	Screening 1	*JAK2*	16	R683S	Missense	TK1^
B-ALL_045	32	AYA	Screening 2	*JAK2*	16	R683S	Missense	TK1
B-ALL_092	16	AYA	Screening 2	*JAK2*	16	R683G	Missense	TK1
GQW_182	48	Adults	Screening 1	*JAK2*	16	R683G	Missense	TK1
GQW_235	37	Adults	Screening 1	*JAK2*	16	R683S	Missense	TK1
GQW_184	51	Adults	Screening 1	*JAK2*	16	R683G	Missense	TK1^
GQW_185	49	Adults	Screening 1	*JAK2*	16	R683G	Missense	TK1^
B-ALL_028	47	Adults	Screening 2	*JAK2*	16	R683S	Missense	TK1
GQW_255	31	AYA	Screening 1	*IL7R*	5	S185C	Missense	Extracellular
B-ALL_073	27	AYA	Screening 2	*IL7R*	6	LTIS -> PRC (243..246)	Inframe indel	TM^
GQW_188	44	Adults	Screening 1	*IL7R*	5	S234N	Missense	Extracellular
GQW_181	44	Adults	Screening 1	*IL7R*	6	DPILLTIS -> DRGC (239..246)	Inframe indel	TM
GQW_186	66	Adults	Screening 1	*IL7R*	6	LLTI -> C (242..245)	Inframe deletion	TM
GQW_198	42	Adults	Screening 1	*IL7R*	6	LT -> LMVKGSFNICG (244)	Inframe insertion	TM
B-ALL_03	40	Adults	Screening 2	*IL7R*	6	ILLTIS -> MC (241..246)	Inframe indel	TM^
B-ALL_013	48	Adults	Screening 2	*IL7R*	6	LTIS -> QKGEC (243..246)	Inframe indel	TM
B-ALL_07	31	AYA	Screening 2	*CRLF2*	6	F232C	Missense	TM
B-ALL_061	27	AYA	Screening 2	*CRLF2*	6	F232C	Missense	TM^
B-ALL_028	47	Adults	Screening 2	*CRLF2*	6	F232C	Missense	TM^
B-ALL_060	21	AYA	Screening 1	*JAK1*	14	A634D	Missense	PK1
GQW-195	43	Adults	Screening 1	*JAK1*	16	R724C	Missense	PK1

### Outcome of B-NEG ALL stratified for age cohorts

To assess whether the mutations identified had a role on patients' outcome, we evaluated the impact on OS and DFS of RAS/RTK and JAK/STAT pathway mutations. Provided the relatively low incidence of the single gene mutations, clonal mutations (detected in >20% of tumor population) were grouped into the JAK/STAT (*IL7R*, *JAK2*, *JAK1*, *CRLF2* mutations and *CRLF2*-rearrangements) and RAS/RTK (*FLT3*, *KRAS*, *NRAS*) pathways. Among the latter, only the most common hot-spot mutations - namely, ITD and D835 *FLT3* mutations and those targeting the G12-13 aminoacidic residues of *KRAS* and *NRAS* - were considered [37, 38]. When analyzing the impact of mutations targeting one pathway, samples harboring mutations of the other pathway were filtered out from the WT cohort.

**Figure 5:**
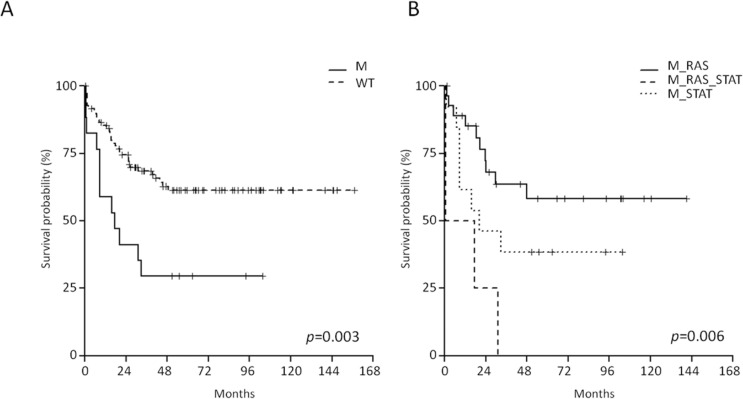
Impact of JAK/STAT pathway mutations on B-NEG ALL patients' outcome **A.** OS of JAK/STAT-mutated *vs* WT cases (analysis on the entire study population). **B.** OS of JAK/STAT-mutated *vs* RAS/RTK-mutated *vs* double mutant cases (analysis on the entire study population).

Firstly, the entire B-NEG cohort - comprising children, AYA and adults - was analyzed and we observed that the RAS/RTK pathway mutations did not impact on long-term survival. At variance, the OS of JAK/STAT-mutated cases was significantly shorter than that of WT cases (29.4% *vs* 62.8% at 4 years; 95% CI 14.1-61.4 and 53.3-73.9, *p* = 0.003) (Figure 5A).

Samples carrying mutations in both pathways (*N* = 4) experienced the worse OS when compared to the samples harboring only JAK/STAT or RAS/RTK (*p* = 0.006); in fact, all the events occurred within 30 months (Figure 5B).

Given the diverse distribution of mutations and the heterogeneous treatments across age cohorts, we repeated these analyses by grouping AYA and adults, whose mutational profile and therapeutic regimens were comparable. This approach led to the results described below.

#### Impact of RAS/RTK pathway mutations on B-NEG ALL patients' outcome

**Figure 6:**
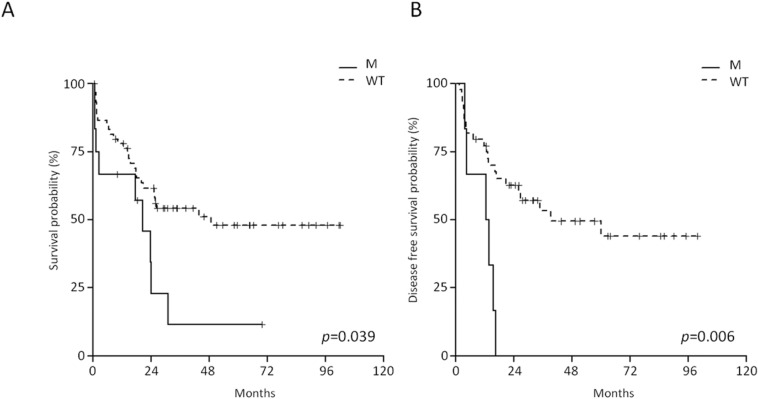
Impact of *RAS* mutations on the outcome of adults and AYA B-NEG ALL patients **A.** OS of *RAS*-mutated *vs* WT cases. **B.** DFS of *RAS*-mutated *vs* WT cases.

Among the RAS/RTK pathway members, the mutations targeting *KRAS*/*NRAS* hotspot exerted the strongest effect on outcome. AYA and adult B-NEG ALL patients harboring *RAS* mutations displayed a shorter OS at 4 years than WT cases (11.4% *vs* 50%, CI 95% 1.8-70.8 and 37.8-66.2, *p* = 0.039) (Figure 6A). *RAS*-mutated cases had also a significantly shorter disease-free survival (DFS) (*p* = 0.006), being 0 at 4 years compared to 47.8% (95% CI 33.5-68.3) for WT cases (Figure 6B). On multivariate analysis, *RAS* mutations and age retained their significance on OS (*p* = 0.0171 and *p* = 0.0002, respectively) and DFS (*p* = 0.0458 and *p* = 0.0128, respectively).

Contrariwise, in the pediatric cohort the survival of RAS/RTK-mutated cases resembled that observed in WT cases.

#### Impact of JAK/STAT mutations on B-NEG ALL patient's outcome

**Figure 7:**
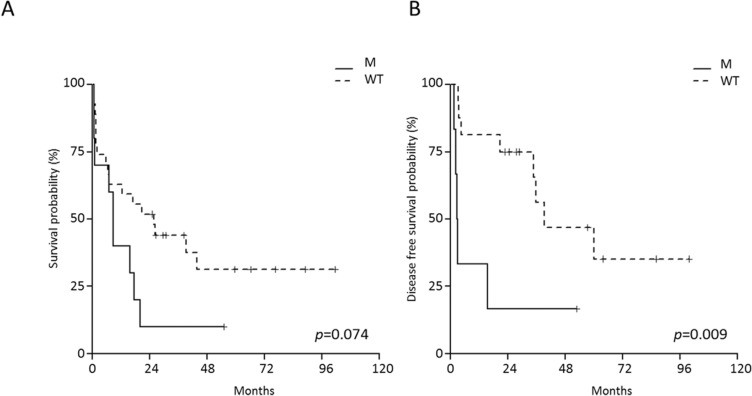
Impact of JAK/STAT mutations on the outcome of adult B-NEG ALL patients **A.** OS of JAK/STAT-mutated *vs* WT. **B.** DFS of JAK/STAT-mutated *vs* WT cases.

The evaluation of the impact of JAK/STAT mutations on B-NEG ALL patient's outcome was confined to AYA and adult cases, since these mutations were absent in children. This analysis documented the negative impact of JAK/STAT mutations on the OS of adult patients only, with JAK/STAT-mutated cases having a 10% (95% CI 1.6-64.2) survival probability at 4 years, as opposed to 31.3% (95% CI 16.6-59.2) for WT patients (Figure 7A). JAK/STAT-mutated cases also had a DFS at 4 years of 16.7% (95% CI 2.8-99.7) compared to 46.9% (95% CI 25.6-86) for WT patients (*p* = 0.009, Figure 7B).

On multivariate analysis, JAK/STAT pathway mutations and WBC retained their significance on DFS (*p* = 0.0259 and *p* = 0.0273, respectively).

### *In vitro* sensitivity assays show the efficacy of target specific inhibition

Finally, in order to examine the potential of targeting the RAS/RTK and JAK/STAT pathways in B-NEG ALLs, we performed *in vitro* sensitivity experiments by treating primary cells from cases harboring known mutations with gene or pathway specific inhibitors.

**Figure 8:**
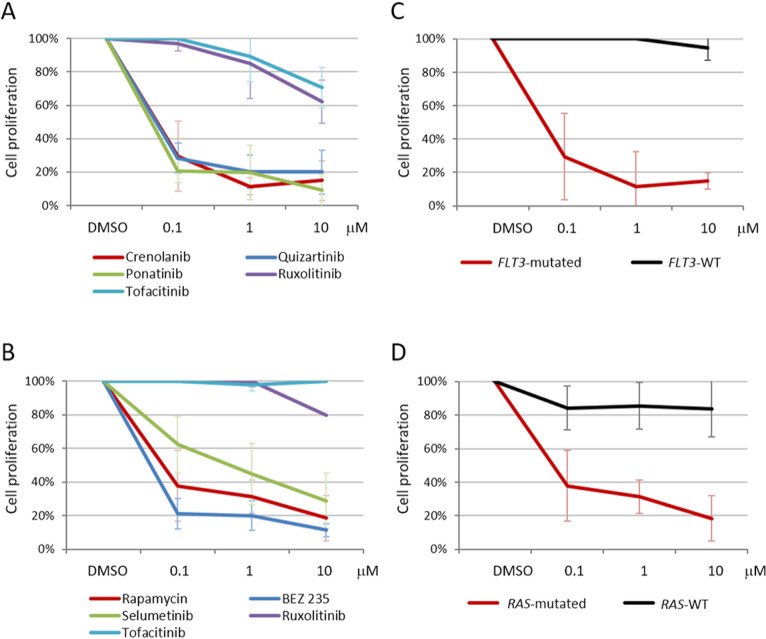
Target-specific inhibitors are effective in reducing B-NEG ALL primary cell proliferation **A.** Average values of ^3^H-thymidine incorporation of primary B-NEG ALL cells from 4 *FLT3*-mutated samples after 72 hours of treatment; crenolanib (0.1 μM) reduced the percentage of proliferating cells to 29.5±21.1%, quizartinib (0.1 μM) to 28.3±9.0% and ponatinib (0.1 μM) to 20.7±7.0%, on average. Compounds not targeting FLT3 (ruxolitinib and tofacitinib) proved ineffective. **B.** Average values of ^3^H-thymidine incorporation of primary B-NEG ALL cells from 4 samples carrying *KRAS* or *NRAS* mutations after 72 hours of treatment; BEZ235 (0.1 μM) reduced the percentage of proliferating cells to 21.3±9.0%, rapamycin (0.1 μM) to 37.8±21.1% and selumetinib to 62.4±16.9%, on average. Compounds not targeting PI3K/mTOR or MEK signaling (ruxolitinib and tofacitinib) proved ineffective. Each condition was run in triplicate. **C.** Average values of ^3^H-thymidine incorporation of primary B-NEG ALL cells from 4 *FLT3*-mutated and 4 *FLT3*-WT samples after 72 hours of treatment with crenolanib (0.1 μM). **D.** Average values of ^3^H-thymidine incorporation of primary B-NEG ALL cells from 4 *RAS*-mutated and 3 *RAS*-WT samples after 72 hours of treatment with rapamycin (0.1 μM).

The FLT3 inhibitors quizartinib and crenolanib and the pan-TK inhibitor ponatinib (0.1 μM) were able to reduce the proliferation rate of primary B-NEG ALL cells from 3 patients carrying *FLT3* ITD mutations and 1 harboring a TKD mutation: indeed, after 72 hours of treatment, quizartinib reduced the percentage of proliferating cells to 28.3±9%, crenolanib to 29.5±21.1% and ponatinib to 20.7±7% (Figure 8A). Primary cells from 4 samples harboring *RAS* mutations - targeting the G12-13 hotspot - were sensitive to PI3K/mTOR inhibitors (0.1 μM): in particular, BEZ235 reduced the percentage of proliferating cells to 21.3±9% and rapamycin to 37.8±21.1%; in addition, cell proliferation decreased to 62.4±16.9% upon treatment with the MEK inhibitor selumetinib (0.1 μM) (Figure 8B). As expected, ruxolitinib and tofacitinib - not targeting FLT3 nor PI3K/mTOR or MEK signaling - proved ineffective in reducing the proliferation of *FLT3* as well as *KRAS/NRAS*-mutated primary cells.

When the sensitivity of primary cells carrying JAK/STAT pathway mutations was tested, ruxolitinib (0.1 μM) proved effective in reducing to 59% the proliferation rate of *JAK2*-mutated cells from 1 case and rapamycin (0.1 μM) reduced to 35.4% the proliferation of *IL7R*-mutated primary cells from another patient, in line with a downstream PI3K/mTOR cascade activation.

Beside their anti-proliferative activity, the above-mentioned drugs also exerted a pro-apoptotic effect, particularly evident for FLT3 inhibitors.

To assess whether the response to the selected inhibitors was specifically associated to the mutational status of B-ALL primary cells, we compared it with B-ALL cases that proved negative for *FLT3*, *KRAS* and *NRAS* mutations. The reduction of proliferation induced by crenolanib, quizartinib and ponatinib (0.1 μM) was significantly higher in *FLT3*-mutated than in *FLT3*-WT cases (*N* = 4, *p* = 0.002, *p* < 0.0001, *p* = 0.0004, respectively) (Figure 8C). Similarly, the proliferation rate of *RAS*-mutated cases was considerably reduced in response to rapamycin and selumetinib (0.1 μM) when compared to *RAS*-WT B-ALL samples (*N* = 3, *p* = 0.024, *p* = 0.037) (Figure 8D). At variance, BEZ235, which simultaneously targets mTOR and PI3K, was also partly active in reducing the proliferation (50.5±21.4%, 0.1 μM) of *RAS*-WT primary cells.

## DISCUSSION

We describe the first in-depth genomic analysis of a broad cohort of B-ALLs lacking known fusion transcripts, including pediatric, AYA and adult cases. This “genetically orphan” subgroup deserves special attention, since it constitutes 70.5% of childhood, 72.7% of AYA and 42.7% of adult ALL cases [3], represents a suitable setting to identify additional lesions sustaining the heterogeneous clinical scenario between children and adults, and reflects the clinical outcome observed in ALL, where the worse prognosis is observed in adults.

By WES of the discovery panel we found that the load of somatic mutations in B-NEG ALL resembles what observed in other leukemias, being 10.5 per sample [26, 28, 39]. Since few recurrent mutations were detected, the study was broadened to two screening panels, for a total of 168 B-NEG ALL samples. This analysis, focused on the genes commonly altered in ALL, revealed an enrichment of mutations targeting genes involved in B-cell development and protein kinases signaling [25-29].

Among the B-cell development genes, *PAX5* mutations were the most common event - occurring in 19% of cases - and increased with age. When *PAX5* deletions were also considered, we observed the same pattern, with 44.6% of adults and only 13.3% of children carrying *PAX5* genetic lesions. Remarkably, similar differences were found when the analysis of CNAs was extended to other B-cell development genes and cell-cycle genes commonly affected by deletions in B-ALL that tend to accumulate with age. Taken together, these results indicate that the genetic background of children is considerably different from patients older than 15 years old.

When focusing on protein kinases cascades, we found that 26.8% of B-NEG ALL cases carry mutations in the RAS/RTK pathway. The deregulation of this pathway may be due to alterations of upstream signaling molecules, like the receptor tyrosine kinase FLT3, or of essential players of the pathway like NRAS and KRAS, as occurred in our series [37].

Within B-ALL, the role of *FLT3*, *KRAS* and *NRAS* mutations has been investigated mostly in the pediatric setting and has been linked to hyperdiploid, *MLL*-rearranged ALL, hypodiploid, as well as high-risk pediatric cases, as elucidated by NGS [26, 30-36]. Our findings encourage a thoughtful reconsideration of their role since we documented that *FLT3*, *KRAS* and *NRAS* mutations are also a common event in B-NEG ALL with a different distribution across age cohorts. In particular, *FLT3* mutations were specific of AYA and adults, while *NRAS* and *KRAS* alterations were prominent in children: in fact, in the latter cohort they collectively accounted for 30% of cases, consistent with the previously reported association with younger age at presentation [36]. Importantly, RAS/RTK mutations were not confined to hyperdiploid cases, as previously reported: in fact, in our cohort mutations were detected in both hyperdiploid and non-hyperdiploid cases.

The clinical impact of RAS/RTK pathway mutations has been explored with contradictory results and seems to be mainly subgroup-dependent. In fact, while several studies, including the largest analysis performed in pediatric cases [36], failed to document any significant effect of *RAS* mutations on survival, a recent paper focusing on *MLL*-rearranged infant ALL documented an independent prognostic factor of *NRAS/KRAS* [33, 35, 36]. Similarly, while the impact of *FLT3* mutations has been widely explored in acute myeloid leukemia [40], it is unclear whether *FLT3* mutations affect the outcome of ALL patients [26, 30, 31]. In our series, given the low number of mutations affecting each single gene we grouped them according to their pathway. When we evaluated the outcome of patients carrying RAS/RTK mutations, we observed a peculiar pattern: AYA and adult mutated cases - all enrolled in clinical trials for adults - had a significantly reduced OS and DFS when compared to WT patients, while the outcome of pediatric cases was not influenced by these mutations, in line with previous reports, most likely because of the effect of more aggressive regimens.

Overall, these results prompted us to investigate the efficacy of specific inhibitors, in light of their availability. Among the *FLT3* inhibitors, the newly introduced quizartinib and crenolanib are promising against ITD and D835 mutations [41]. In our *in vitro* assays, both compounds, as well as the third-generation pan-TKI ponatinib were effective in reducing the proliferation of *FLT3*-mutated primary cells.

Since RAS proteins have proven challenging to inhibit, an alternative strategy is to interfere with the RAS signaling at different levels of the pathway [42, 43]. Encouraging results have been obtained with the inhibitors of PI3K/mTOR and MEK1/2 [26, 44]. Consistently, in the current study, samples carrying *NRAS* or *KRAS* mutations proved highly sensitive to rapamycin and to the dual PI3K/mTOR inhibitor BEZ235; the proliferation rate was also inhibited by the MEK1/2 inhibitor selumetinib, though at a lower extent.

These findings qualify FLT3, PI3K/mTOR and MEK1/2 inhibitors as alternative therapeutic agents for B-NEG ALL patients, especially in adults/elderly who frequently suffer of comorbidities and often cannot undergo intensive chemotherapeutic approaches.

The other set of mutations that captured our attention were those leading to the JAK/STAT signaling activation, being druggable and frequently altered in other poor prognosis ALL subsets [23-25, 34, 45-47]. Overall, we detected JAK/STAT mutations in 12.5% of B-NEG cases and, importantly, only AYA and adults were affected, with an incidence of 14.7% and 21%, respectively. This skewed distribution of lesions might be ascribed to the characteristics of our study population: in fact, unlike previously published papers describing high-risk and *BCR-ABL1*-like pediatric ALL cases [23-25, 34, 45-47], our cohort was not chosen for unfavorable clinical features nor for a peculiar GEP, and included all age cohorts. Therefore, our data indicate that unselected pediatric B-ALLs are rarely affected by these mutations, whereas their incidence increases with age. We provide direct evidence that considering only the mutational status of JAK/STAT members, regardless of GEP and concomitant rearrangements, it is possible to single out adult B-NEG ALLs experiencing a worse OS and DFS. As a matter of fact, the cohort of JAK/STAT-mutated only partly overlap with the *BCR-ABL1*-like profile, thus indicating that the mutational characterization may integrate the identification of *BCR-ABL1*-like cases, presently not completely elucidated nor straightforward.

The identification of JAK/STAT lesions bears also important therapeutic implications given the possibility of interfering with JAK members or downstream pathways as shown by previous experimental models [42, 43, 48] and our pilot experiments on primary cells.

In conclusion, our study, that was carried out in B-ALL cases without major molecular rearrangements of all ages, implements the current knowledge on ALL. Firstly, we hereby show that the genetic background is significantly different between children, AYA and adults, with the number of deleterious lesions incrementing with age progression; this phenomenon might, at least in part, account for the different outcome between age cohorts. Second we show that RAS/RTK pathway mutations negatively impact on OS and DFS while they do not affect children's outcome. Along the same line, we show that JAK/STAT mutations confer a worse prognosis within the adult population. Finally, we show that by targeting these lesions we can reduce the *in vitro* leukemic cell proliferation, thus indicating that specific inhibitors might be integrated in the therapeutic backbone of ALL.

## MATERIALS AND METHODS

### Experimental strategy

Bone marrow samples from 168 patients (median age: 23 years, range 1.2-78) with newly diagnosed B-ALL, containing >70% of leukemic blasts and negative for recurrent fusion genes (*BCR-ABL1*, *ETV6-RUNX1*, *E2A-PBX1*, *MLL* rearrangements, defined B-NEG), were included in the present research. Three age cohorts were considered: 1-15 years (children, 50 patients); 16-35 years (AYA, 61 patients); >36 years (adults, 57 patients). The study involved 3 phases (Figure S5): 1) WES and CNA analysis of 13 samples and their paired germline DNA (discovery panel, Table S4); 2) WES of 68 additional cases (screening panel 1, Table S5); 3) sequencing of selected genes by Sanger or NGS in 87 patients (screening panel 2, Table S5). Further details are provided in Supplemental Material and Table S6.

### Sequencing analyses

Tumor and germline genomic DNA (gDNA, 3 μg) from the discovery panel patients and gDNA (1 μg) from the screening panel 1 tumor samples were enriched in protein coding sequences by the SureSelect Human All Exon 50Mb and the SureSelectXT2 Human All Exon kits, respectively (Agilent Technologies, Santa Clara, CA). Captured targets were subjected to massively parallel sequencing (HiSeq 2000 analyzer, Illumina, San Diego, CA) with the paired-end 2 × 100 bp read option (Tables S7 and S8).

Mapping to the hg19/NCBI GRCh37 and identification of tumor-specific variants of the discovery panel was performed as described [39, 49], while the analysis of screening panel 1 required more stringent criteria (Supplemental Material).

After validation by Sanger, frequently mutated genes (*PAX5*, *FLT3*, *KRAS*, *NRAS, JAK2, JAK1, IL7R, CRLF2*) were sequenced in the screening panel 2 by Sanger sequencing or NGS (Genome Sequencer Junior 454, Roche Applied Science®) (Supplemental Material, Table S9).

### CNA and GEP analyses

CNA analysis of the discovery panel was performed with the Genome-Wide Human SNP Array 6.0 (Affymetrix, Santa Clara, CA; GSE67405) and processed as previously described [39]. Recurrent deletions and *CRLF2* rearrangements were screened by the Salsa MLPA P335-A3 ALL-IKZF1 kit (MRC-Holland, Amsterdam, the Netherlands) and analyzed as reported [50, 51].

GEP of B-NEG ALLs carrying JAK/STAT mutations was performed by using the HGU133 Plus 2.0 gene chips (Affymetrix) according to manufacturer's instructions and GEP analyses were carried out by dChip software (http://www.hsph.harvard.edu/cli/complab/dchip/). In order to recognize *BCR-ABL1*-like cases, gene expression levels of a set of genes specific of the *BCR-ABL1*-like profile, selected in the context of a previously described dataset [52], were evaluated in JAK/STAT-mutated cases.

### Statistical analyses

Patients' characteristics were compared by chi-squared or Fisher's exact test for categorical variables. Differences in distributions were assessed by Wilcoxon test for continuous data. OS and DFS curves were estimated by Kaplan-Meier product-limit method and compared using the log-rank test. Multivariate analysis was performed with Cox proportional hazards regression model to adjust the effect of *RAS* and JAK/STAT pathway mutations for WBC and platelets counts, hemoglobin levels, age and gender, on OS and DFS. All tests were 2-sided, accepting *p* < 0.05 as index of statistical significance. All analyses relied on SAS v9.4 software.

### *In vitro* assays

Annexin V/7AAD apoptotic test (BD Bioscience, San Josè, CA), MTT (TOX1 assay kit, Sigma Aldrich, St Louis, MO) and ^3^H-thymidine (Perkin Elmer, Waltham, MA) proliferation assays were performed to assess the sensitivity of primary B-NEG ALL cells carrying mutations of *FLT3*, *KRAS*, *NRAS*, *IL7R*, *JAK2* or WT for the same mutations to increasing doses (0.1-10 μM) of selected inhibitors (Selleck Chemicals Houston, TX) - of tyrosine kinases (ponatinib), *FLT3* (quizartinib, crenolanib), PI3K/mTOR/MEK pathway (rapamycin, BEZ235, selumetinib) and *JAK1/2* (tofacitinib, ruxolitinib). Drugs were added at time 0 and viability was measured after 48 and 72 hours. Cells were plated at 1×10^6^/ml and each condition was run in triplicate.

## SUPPLEMENTAL MATERIALS AND METHODS TABLES AND FIGURES


