# Supplementary material for: Prognostic and therapeutic role of targetable lesions in B-lineage acute lymphoblastic leukemia without recurrent fusion genes
Source: Oncotarget. 2016 Feb 12;7(12):13886–901. doi: 10.18632/oncotarget.7356 (PMC4924686; doi:10.18632/oncotarget.7356)

Prognostic and therapeutic role of targetable lesions in B-lineage acute lymphoblastic leukemia without recurrent fusion genes

SUPPLEMENTAL MATERIALS AND METHODS

Description of the study population

Overall, 168 patients (102 patients were males and 66 females, median age: 23 years, range 1.2-78) were included in the present study and were divided in 3 age cohorts. Patients were enrolled in different GIMEMA (Gruppo Italiano Malattie EMatologiche dell'Adulto) and AIEOP (Associazione Italiana Ematologia Oncologia Pediatrica) protocols, designed for adult and pediatric patients, respectively (Table S6). The study was approved by the local IRB and all patients or tutors gave their informed consent to the biological analyses in accordance with the Declaration of Helsinki.

Clinico-biological features of our cohort did not differ from the population enrolled in the above-mentioned protocols, apart from a higher white blood cell (WBC) count (p<0.0001), that, however, did not entail a different overall survival (OS, p=0.56) and disease-free survival (DFS, p=0.62).

Sequence mapping and identification of tumor-specific variants of the discovery panel

Paired-end reads obtained by high-throughput sequencing of the discovery panel were aligned to the human genome reference hg19/NCBI GRCh37 using the BWA alignment tool version 0.5.9. Small sequence variants, i.e. single nucleotide variants and small insertion/deletions (indels), were identified separately for each tumor and germline sample. The allele frequency of each variant was estimated from the number of mutated reads and the total number of reads covering the position of that variant. Using the SAVI algorithm [1], an empirical prior was constructed based on

the data of each sample, from which we obtained a corresponding high-credibility interval (posterior probability ≥99.9%) for the frequency of each variant and a high-credibility interval for the corresponding change in frequency between the tumor and the normal samples. The non-silent variants, not reported as common dbSNP, with allele frequency ≥20% in tumor and ≤1% in normal, and with significant change in allele frequency between tumor and the normal ($p<1e^{-5}$), were retained as somatic variants.

In silico analysis of the mutated genes

Mutations were mainly missense (94%) and only marginally in-frame (3%) or frameshift deletions (3%). Genes resulted mutated from WES of the discovery and screening panel 1 were assigned to functional categories and pathways by means of DAVID software (http://david.abcc.ncifcrf.gov/) and the Molecular Signatures Database (http://www.broadinstitute.org/gsea/msigdb/index.jsp) [2, 3]. Also, it was verified whether they were listed in the Catalogue of Somatic Mutations in the Cancer database and in the Cancer Gene Census database (http://www.sanger.ac.uk/genetics/CGP/Census/) [4].

Lastly, confirmed, somatic non-silent mutations were tested for their consequences by the PolyPhen-2 algorithm (http://genetics.bwh.harvard.edu/pph2/) [5].

Sequence mapping and identification of tumor-specific variants of the screening panel 1

Similar to the analysis in the discovery panel, pair-end reads from WES of the screening panel 1 were aligned to hg19 by BWA. High confident SNVs and small indels were then selected by SAVI algorithm. Due to lack of normal control, more stringent criteria were required to eliminate sequencing artifacts and genetic polymorphisms. Only the non-silent variants, not reported as

common dbSNP and in large collection of normal samples from The Cancer Genome Atlas (TCGA), with allele frequency >20% average mapping quality >30, and with at least 2 reads in both forward and backward strands were kept for further study. Based on selected variants, we further integrated the information from the Catalog of somatic mutations (COSMIC), the gene length, and recurrence in different cohorts to identify potential driver genes.

Validation of candidate somatic mutations by Sanger sequencing

The sequences surrounding the genomic locations of the candidate tumor-specific non-silent mutations were obtained from the UCSC Human Genome database (http://genome.ucsc.edu/), and PCR primers were derived from previously published studies [6] or were custom-designed using the Primer 3 online software (http://frodo.wi.mit.edu/primer3/) and the *in silico* PCR tool of the UCSC Human Genome database to verify the uniqueness of the match. Primers sequences are available upon request.

Screening of recurrently mutated genes by Sanger sequencing

The coding exons and splice sites of *FLT3*, *KRAS*, *NRAS, JAK2* (exons 20, 21) and *JAK1* (exons 14-21) were analyzed by PCR amplification and Sanger sequencing of the whole-genome-amplified DNA generated by the Repli-g Mini kit (Qiagen, Hilden, Germany). PCR primers were designed as described above and available upon request. Sequence analysis was performed by using the Mutation Surveyor Version 3.97 software (SoftGenetics, State College, PA) and, after subtracting synonymous mutations and SNPs, the remaining candidate somatic mutations were confirmed on high-molecular-weight genomic DNA from both tumor and normal (when available) samples by performing PCR amplification and bidirectional direct sequencing.

Screening of recurrently mutated genes by NGS

Hotspot mutations targeting *PAX5*, *JAK2*, *IL7R*, *CRLF2* were screened by using the Genome Sequencer Junior 454 (Roche Applied Science®) and specific primers and MIDs (Table S9).

Raw images and internal control analysis of each run were performed using settings of the GS Run Browser Software version 2.3 (Roche Applied Science®). A cut-off of 80 reads in forward and in reverse (combined n=160 reads/amplicon/sample) was established as pass filter for subsequent variants analysis. The Sequence Pilot software version 3.4.0 (JSI Medical System, Kippenheim, Germany) was used for sequence analyses and variants detection. Mutations within introns, synonymous mutations and SNPs were filtered out. The mutational load was expressed as mutated allele frequency (MAF) based on the percentage of mutated reads. Only variants above 2% both in forward and in reverse reads were retained.

Screening of recurrently deleted genes

The copy number status of the genes commonly deleted (i.e. *IKZF1*, *CDKN2A/2B*, *PAX5*, *EBF1*, *ETV6*, *BTG1*) in B-ALL was assessed by multiplex ligation-dependent probe amplification (MLPA) on 157/168 samples, belonging to the screening panels 1 and 2, using the Salsa MLPA P335-A3 ALL-IKZF1 kit (MRC-Holland, Amsterdam, the Netherlands) and manufacturers' recommendations. Genomic DNA from at least 3 healthy donors per experiment was used as wild-type (WT) control. Data analysis was performed by Coffalyser.Net software (www.coffalyser.net) and in agreement with previously published papers [7].

<u>Supplemental Results</u>

<u>*In silico* analyses of the mutated genes</u>

In silico analyses showed that all the genes affected by mutations were mentioned in COSMIC and 11 (*ARID1A*, *FGFR3*, *FLT3*, *KRAS*, *NF1*, *NFKB2*, *PAX5*, *PDGFRB*, *PHOX2B*, *SETD2*, *SS18L1*) were also listed in the Cancer Gene Census database as being involved in oncogenesis, thus suggesting a role also in the pathogenesis of B-ALL. Interestingly, when a functional annotation analysis was run by DAVID software, we found a significant enrichment of genes belonging the following categories: Focal adhesion/ECM interaction, Small GTPase mediated signal transduction, Ion transport and Protein kinase activity (Table S2).

REFERENCES

1. Trifonov V, Pasqualucci L, Tiacci E, Falini B, Rabadan R. SAVI: a statistical algorithm for variant frequency identification. BMC Syst Biol. 2013; 7 Suppl 2: S2.
2. Huang da W, Sherman BT, Lempicki RA. Systematic and integrative analysis of large gene lists using DAVID bioinformatics resources. Nat Protoc. 2009; 4(1): 44-57.
3. Subramanian A, Tamayo P, Mootha VK, Mukherjee S, Ebert BL, Gillette MA, Paulovich A, Pomeroy SL, Golub TR, Lander ES, Mesirov JP. Gene set enrichment analysis: a knowledge-based approach for interpreting genome-wide expression profiles. Proc Natl Acad Sci U S A. 2005; 102(43): 15545-50.
4. Forbes SA, Bindal N, Bamford S, Cole C, Kok CY, Beare D, Jia M, Shepherd R, Leung K, Menzies A, Teague JW, Campbell PJ, Stratton MR, Futreal PA. COSMIC: mining complete cancer genomes in the Catalogue of Somatic Mutations in Cancer. Nucleic Acids Res. 2011; 39 (Database issue): D945-50.
5. Adzhubei IA, Schmidt S, Peshkin L, Ramensky VE, Gerasimova A, Bork P, Kondrashov AS, Sunyaev SR. A method and server for predicting damaging missense mutations. Nat Methods. 2010; 7(4): 248-9.
6. Parsons DW, Li M, Zhang X, Jones S, Leary RJ, Lin JC, Boca SM, Carter H, Samayoa J, Bettegowda C, Gallia GL, Jallo GI, Binder ZA, et al . The genetic landscape of the childhood cancer medulloblastoma. Science. 2011; 331(6016): 435-9.
7. Palmi C, Lana T, Silvestri D, Savino A, Kronnie GT, Conter V, Basso G, Biondi A, Valsecchi MG, Cazzaniga G. Impact of IKZF1 deletions on IKZF1 expression and outcome in Philadelphia chromosome negative childhood BCP-ALL. Reply to "incidence and biological significance of IKZF1/Ikaros gene deletions in pediatric Philadelphia chromosome negative and Philadelphia chromosome positive B-cell precursor acute lymphoblastic leukemia". Haematologica. 2013; 98(12): e164-5.

Table S1. Somatic mutations identified by WES in the B-NEG ALL discovery panel.

Sample ID	Gene	CCDS ID	Exon^	Chr	Start^^	End^^	Ref nt	Var nt	T frequency	Mutation Type	AA change	PolyPhen-2 (score)
ALL-1	AKAP8	CCDS12329.1	5	19	15484077	15484077	C	G	23	Missense	S149T	po D (0.534)
ALL-1	APOBEC3B	CCDS13982.1	2	22	39380212	39380212	G	A	55	Nonsense	W50*	nd
ALL-1	CNTNAP5	CCDS46401.1	19	2	125555682	125555682	C	T	49	Missense	A1000V	pr D (1)
ALL-1	EXPH5	CCDS8341.1	6	11	108382098	108382098	G	A	45	Missense	S1379L	B (0.054)
ALL-1	FGFR3	CCDS3353.1	8	4	1806233	1806233	C	T	45	Missense	P418S	pr D (1)
ALL-1	G6PC	CCDS11446.1	5	17	41063352	41063352	G	A	42	Missense	C328Y	pr D (1)
ALL-1	GABRA5	CCDS45194.1	3	15	27128367	27128367	C	T	45	Missense	S88F	pr D (1)
ALL-1	LGR5	CCDS9000.1	15	12	71972615	71972615	C	T	61	Missense	P438S	pr D (1)
ALL-1	LPHN2	CCDS689.1	10	1	82432183	82432183	C	T	53	Missense	R730C	pr D (0.998)
ALL-1	MAP1B	CCDS4012.1	5	5	71496087	71496087	C	T	43	Missense	P2302L	B (0.002)
ALL-1	MDN1	CCDS5024.1	60	6	90406289	90406289	G	A	25	Missense	P3058L	pr D (0.998)
ALL-1	MVD	CCDS10968.1	5	16	88722658	88722658	C	G	31	Missense	R153P	pr D (1)
ALL-1	MYH13	CCDS45613.1	36	17	10206803	10206803	C	T	40	Missense	E1827K	pr D (1)
ALL-1	MYO6	CCDS34487.1	33	6	76623969	76623969	C	T	27	Missense	P1210L	pr D (1)
ALL-1	NFKB2	CCDS41564.1	8	10	104157768	104157768	C	T	31	Missense	S231F	pr D (1)
ALL-1	PTRF	CCDS11425.1	2	17	40557056	40557056	C	T	37	Missense	M274I	B (0.005)
ALL-1	SLC5A12	CCDS7860.2	5	11	26725413	26725413	C	T	59	Missense	G203R	pr D (0.998)
ALL-1	TAAR2	CCDS5157.1<br>CCDS34541.1	1,2	6	132938923	132938923	C	T	23	Missense	R96K,R141K	pr D (1)
ALL-1	VCAN	CCDS4060.1<br>CCDS47242.1	14,12	5	82876248	82876248	C	T	43	Missense	R3396C,R655C	pr D (1)
ALL-2	ALDH1A2	CCDS10163.1<br>CCDS10164.1<br>CCDS45266.1	9,8,7	15	58256129	58256129	C	T	29	Missense	R347H,R309H, R251H	B (0.026)
ALL-2	ATP5G2	CCDS31812.1<br>CCDS8863.2	3,3	12	54063678	54063678	G	A	32	Nonsense	R48*,R89*	nd
ALL-2	CACNA1G	CCDS45730.1<br>CCDS45733.1<br>CCDS45734.1	27,26,25	17	48692787	48692787	A	C	20	Missense	T1609P, T1591P, T1575P	pr D (1)
ALL-2	CADM2	CCDS33792.1	7	3	86010650	86010650	G	A	35	Missense	G268R	pr D (0.998)
ALL-2	DCAF5	CCDS32106.1	9	14	69520902	69520904	TTG	-	29	In frame deletion	N833- N834-	nd
ALL-2	HOXA6	CCDS5407.1	2	7	27185428	27185428	C	T	28	Missense	R184H	pr D (0.999)
ALL-2	KLF5	CCDS9448.1	4	13	73649980	73649980	C	T	25	Missense	R444C	pr D (0.983)
ALL-2	NPHS1	CCDS32996.1	21	19	36330506	36330506	C	T	27	Missense	R940H	po D (0.517)
ALL-2	SLC24A5	CCDS10128.1	3	15	48426500	48426500	C	T	24	Missense	A116V	B (0.036)
ALL-2	VWDE	CCDS47544.1	12	7	12409638	12409638	G	A	30	Missense	P765L	B (0)
ALL-3	ANO5	CCDS31444.1	21	11	22297675	22297675	A	G	22	Missense	N817S	B (0.009)
ALL-3	C8orf84	CCDS43747.2	2	8	73993367	73993367	C	T	25	Missense	R99H	pr D (1)
ALL-3	COL11A1	CCDS778.1<br>CCDS780.1	40,39	1	103427739	103427739	C	A	32	Missense	G1036V,G997V	pr D (1)
ALL-3	EIF2S3	CCDS14210.1	2	X	24073777	24073777	C	A	37	Missense	Q39K	pr D (1)
ALL-3	IRF2BP1	CCDS12678.1	1	19	46387778	46387778	C	A	31	Missense	V419L	B (0.005)
ALL-3	LILRA2	CCDS46179.1	5	19	55086996	55086996	A	G	31	Missense	D310G	pr D (0.998)
ALL-3	MBTPS1	CCDS10941.1	1	16	84135288	84135288	G	A	41	Missense	P34L	B (0)
ALL-3	OR4D6	CCDS31562.1	1	11	59225212	59225212	G	A	31	Missense	R260Q	po D (0.94)
ALL-3	ZNF217	CCDS13443.1	1	20	52198585	52198599	TCCTGCCGTAA GGAG	CCGCTCT T	33	Frameshift indel	G256- G257- M258- P259- S260- S261- C259+ R260+ A261+	nd
ALL-4	C17orf71	CCDS11615.1	3	17	57290400	57290400	G	A	59	Missense	R739Q	pr D (0.991)
ALL-4	CT47B1	CCDS48161.1	1	X	120009185	120009185	G	A	30	Missense	R114W	pr D (0.996)
ALL-4	DNAH10	CCDS9255.2	69	12	124413087	124413087	G	A	33	Missense	D3969N	B (0.314)
ALL-4	FLT3	CCDS31953.1	20	13	28592635	28592637	ATG	-	51	In frame deletion	I836, M837-	nd
ALL-4	KIAA1755	CCDS33467.1	4	20	36867930	36867930	C	A	28	Missense	G583C	pr D (1)
ALL-4	NYNRIN	CCDS45090.1	8	14	24884755	24884755	A	G	24	Missense	K1267R	B (0.218)
ALL-4	PHOX2B	CCDS3463.1	3	4	41747843	41747843	A	C	50	Missense	V309G	pr D (0.998)
ALL-4	SLC25A46	CCDS4100.1	8	5	110097399	110097399	G	A	42	Missense	V392I	B (0.027)
ALL-5	CCDC78	CCDS32353.1	8	16	774802	774802	A	G	29	Missense	L215P	pr D (1)
ALL-5	FRG2B	CCDS44502.1	4	10	135438836	135438836	G	T	20	Missense	L202I	pr D (0.986)
ALL-5	GNRH2	CCDS13040.1<br>CCDS13041.1<br>CCDS13042.1	3,3,3	20	3026345	3026345	-	GCCCC	25	Frameshift insertion	E109+,E102+, E101+	nd
ALL-5	ITGA11	CCDS45291.1	13	15	68624762	68624762	C	T	48	Missense	V494M	B (0.007)
ALL-5	NHLH2	CCDS885.1	1	1	116380950	116380950	C	A	33	Missense	S15I	pr D (0.984)
ALL-5	OR8B8	CCDS8446.1	1	11	124310957	124310957	C	T	52	Missense	V9M	B (0.088)
ALL-5	PLXNA3	CCDS14752.1	17	X	153695461	153695461	C	T	23	Missense	R1057C	pr D (1)
ALL-5	TADA2B	CCDS47007.1	2	4	7056213	7056213	T	A	27	Missense	F232Y	po D (0.911)

ALL-5	TADA2B	CCDS47007.1	2	4	7056216	7056216	-	CCA	21	In frame insertion	Q234+	nd
ALL-5	TCERG1	CCDS4282.1<br>CCDS43379.1	8,7	5	145850206	145850210	AGAAG	-	52	Frameshift deletion	L469-, E470, E471/L448-, E449, E450	nd
ALL-6	BAI3	CCDS4968.1	28	6	70082310	70082310	C	T	24	Nonsense	R1418*	nd
ALL-6	FAM167A	CCDS5981.1	1	8	11301796	11301796	C	T	25	Missense	R42H	pr D (1)
ALL-6	GPR83	CCDS8297.1	4	11	94113673	94113673	C	A	33	Missense	W305L	pr D (1)
ALL-6	IL1RAPL1	CCDS14218.1	10	X	29973626	29973626	G	A	46	Missense	A594T	B (0.002)
ALL-6	RASGRP2	CCDS31598.1	8	11	64504274	64504274	C	A	36	Missense	S349I	B (0.052)
ALL-6	SS18L1	CCDS13491.1	6	20	60738592	60738592	C	T	27	Missense	S212L	po D (0.465)
ALL-6	ZDHHC14	CCDS5252.1<br>CCDS47510.1	3,3	6	158014145	158014145	G	A	23	Missense	A178T,A178T	po D (0.865)
ALL-7	ABCC2	CCDS7484.1	2	10	101544387	101544387	C	T	43	Missense	P19L	po D (0.839)
ALL-7	AGTR1	CCDS3137.1	1	3	148459240	148459240	C	T	53	Missense	R140C	pr D (1)
ALL-7	AP2A1	CCDS46148.1<br>CCDS46149.1	23,22	19	50309488	50309488	T	A	31	Missense	L945Q,L923Q	pr D (1)
ALL-7	COL6A1	CCDS13727.1	31	21	47421948	47421948	G	A	100	Missense	R677H	B (0.021)
ALL-7	COL6A1	CCDS13727.1	35	21	47423817	47423817	G	A	90	Missense	E993K	po D (0.919)
ALL-7	DCHS2	CCDS3785.1	25	4	155156598	155156598	G	A	49	Missense	P2614L	B (0.071)
ALL-7	EPHA5	CCDS3513.1<br>CCDS3514.1	11,10	4	66231734	66231734	A	G	48	Missense	Y656H,Y634H	pr D (1)
ALL-7	FBN2	CCDS34222.1	30	5	127670915	127670915	C	T	49	Missense	R1307H	B (0.027)
ALL-7	GTF2A1L, STON1-GTF2A1L	CCDS46281.1<br>CCDS1840.1	3,4	2	48848310	48848310	G	T	46	Missense	W43L,W747L	pr D (1)
ALL-7	HLA-B	CCDS34394.1	3	6	31324195	31324195	T	A	42	Missense	Y123F	B (0.004)
ALL-7	ITGA5	CCDS8880.1	27	12	54793500	54793500	C	G	40	Missense	G924R	pr D (1)
ALL-7	LRRC30	CCDS42409.1	1	18	7231198	7231198	C	T	47	Missense	T21M	B (0.071)
ALL-7	SH3GLB2	CCDS6916.1	10	9	131771419	131771419	G	A	57	Missense	A349V	po D (0.875)
ALL-7	TRPC7	CCDS47267.1	5	5	135587487	135587487	C	T	45	Missense	G477R	po D (0.924)
ALL-8	ANKRD20A2	CCDS35028.1	15	9	42409857	42409857	G	T	31	Missense	A616S	pr D (0.989)
ALL-8	ANKRD30A	CCDS7193.1	7	10	37430979	37430979	G	T	42	Missense	W329L	po D (0.659)
ALL-8	COL1A2	CCDS34682.1	49	7	94057162	94057162	G	A	51	Missense	R1164H	pr D (1)
ALL-8	DRG2	CCDS11191.1	5	17	18003009	18003009	C	A	33	Missense	L147M	pr D (1)
ALL-8	FLT3	CCDS31953.1	20	13	28592621	28592621	A	T	72	Missense	Y842N	pr D (1)
ALL-8	GTDC1	CCDS2185.1	1	2	144966273	144966273	C	A	43	Nonsense	G26*	nd
ALL-8	LONRF2	CCDS2046.2	2	2	100925658	100925658	G	A	40	Missense	R237W	pr D (1)
ALL-8	PCDHA11	CCDS47284.1	1	5	140249854	140249854	C	T	29	Missense	T389M	po D (0.83)
ALL-8	PREX2	CCDS6201.1	6	8	68942753	68942753	C	T	54	Missense	R189W	po D (0.912)
ALL-8	SETD2	CCDS2749.2	5	3	47155366	47155366	G	C	49	Missense	S1572W	pr D (0.999)
ALL-8	TBC1D21	CCDS10252.1	8	15	74178904	74178904	G	A	71	Missense	C243Y	B (0.101)
ALL-8	USP32	CCDS32697.1	21	17	58288396	58288396	T	C	29	Missense	N801S	B (0.011)
ALL-8	WWC1	CCDS4366.1	11	5	167850581	167850581	G	A	23	Missense	G440R	pr D (1)
ALL-8	ZNF250	CCDS34972.1	5	8	146106958	146106958	C	T	52	Missense	R542H	pr D (0.989)
ALL-9	CSNK1E	CCDS13970.1	4	22	38696791	38696791	C	T	45	Missense	R168Q	pr D (1)
ALL-9	GCNT4	CCDS4026.1	1	5	74325046	74325046	G	A	28	Missense	R273W	po D (0.946)
ALL-9	GRIN2B	CCDS8662.1	6	12	13768155	13768155	T	A	37	Missense	N516I	po D (0.952)
ALL-9	NF1	CCDS42292.1	12	17	29533315	29533315	C	T	47	Nonsense	R440*	nd
ALL-9	NF1	CCDS42292.1<br>CCDS11264.1	32,31	17	29585494	29585494	G	A	51	Missense	E1436K,E1415K	B (0.024)
ALL-9	PAPSS1	CCDS3676.1	8	4	108575880	108575880	T	C	40	Missense	T358A	po D (0.935)
ALL-10	ADAMTSL2	CCDS6976.1	2	9	136402527	136402527	G	T	23	Missense	D31Y	pr D (0.997)
ALL-10	ANKRD17	CCDS34003.1	1	4	74124123	74124123	C	G	22	Missense	S88T	B (0.14)
ALL-10	ARRB2	CCDS11050.1<br>CCDS11051.1	7,6	17	4620986	4620986	G	T	25	Missense	G142C,G127C	pr D (1)
ALL-10	IRS4	CCDS14544.1	1	X	107977758	107977758	C	T	26	Missense	R606H	B (0.018)
ALL-10	PAX5	CCDS6607.1	3	9	37015165	37015165	G	C	24	Missense	P80R	pr D (0.999)
ALL-10	PRSS23	CCDS8278.1	1	11	86519206	86519206	C	T	28	Missense	A174V	pr D (1)
ALL-10	RASSF1	CCDS43096.1<br>CCDS2820.1	2,2	3	50375431	50375431	T	G	36	Missense	T88P,T92P	po D (0.904)
ALL-10	SLC26A3	CCDS5748.1	12	7	107418675	107418675	C	A	22	Missense	G487C	pr D (1)
ALL-10	TTC39B	CCDS6477.1	3	9	15225928	15225928	A	G	42	Missense	S54P	B (0)
ALL-10	USH1C	CCDS7825.1	5	11	17548863	17548863	C	T	33	Missense	V135I	pr D (1)
ALL-10	WASF3	CCDS9318.1	6	13	27255387	27255387	C	-	39	Frameshift deletion	P305-	nd
ALL-11	AOC2	CCDS45690.1	2	17	41001306	41001306	G	A	31	Missense	G598R	pr D (1)
ALL-11	ARID1A	CCDS44091.1<br>CCDS285.1	20,20	1	27105553	27105553	C	T	30	Nonsense	R1505*,R1722*	nd
ALL-11	CCNE2	CCDS6264.1	2	8	95906312	95906312	C	G	40	Missense	S17T	B (0.018)
ALL-11	CCNL1	CCDS3178.1	5	3	156869993	156869993	C	T	41	Missense	R216H	pr D (0.98)
ALL-11	CNGB1	CCDS42169.1	31	16	57921836	57921836	G	C	47	Missense	L1129V	pr D (0.993)
ALL-11	ETV7	CCDS4819.1	5	6	36339257	36339257	C	A	33	Missense	G172C	pr D (0.957)
ALL-11	GAST	CCDS11404.1	1	17	39871735	39871735	C	T	50	Missense	A16V	pr D (0.999)
ALL-11	HLA-DRB1	CCDS47409.1	2	6	32551912	32551912	A	C	50	Missense	V115G	B (0.001)

ALL-11	LRRC28	CCDS10380.1	4	15	99828121	99828121	G	A	22	Missense	R117Q	pr D (0.991)
ALL-11	PPP2R2C	CCDS3387.1	7	4	6335353	6335353	C	T	36	Missense	R299Q	pr D (0.976)
ALL-13	ADAMTS2	CCDS4444.1	17	5	178555006	178555006	C	A	22	Missense	E857D	pr D (0.982)
ALL-13	CAPRIN1	CCDS31453.1	12	11	34111803	34111803	A	C	32	Missense	Q457H	pr D (0.958)
ALL-13	CSMD3	CCDS6315.1 CCDS6316.2 CCDS6317.1	57,55,57	8	113301605	113301605	G	T	40	Missense	P3046H, P2877H, P3006H	pr D (1)
ALL-13	HLA-C	CCDS34393.1	2	6	31239613	31239613	C	T	20	Missense	V36M	pr D (1)
ALL-13	KRAS	CCDS8702.1	3	12	25378562	25378562	C	T	45	Missense	A146T	pr D (0.993)
ALL-13	PAX5	CCDS6607.1	3	9	37015165	37015165	G	C	83	Missense	P80R	pr D (0.999)
ALL-13	SLC1A6	CCDS12321.1	1	19	15083563	15083563	G	A	31	Missense	R54C	B (0.063)
ALL-14	CDC27	CCDS45720.1 CCDS11509.1	11,11	17	45219676	45219676	C	A	24	Missense	D439Y,D433Y	pr D (0.989)
ALL-14	EGFLAM	CCDS3924.1 CCDS3925.1	8,3	5	38407026	38407026	A	T	50	Missense	I309F,I75F	B (0.002)
ALL-14	HLA-DRB5	CCDS4751.1	1	6	32497968	32497968	T	A	24	Missense	M12L	po D (0.802)
ALL-14	HLA-DRB5	CCDS4751.1	1	6	32497971	32497971	A	T	25	Missense	Y11N	B (0.027)
ALL-14	KCTD19	CCDS42179.1	6	16	67333416	67333416	C	A	36	Missense	S279I	B (0.154)
ALL-14	KIAA1614	CCDS41442.1	2	1	180885903	180885903	G	A	42	Missense	G222R	B (0.004)
ALL-14	MAST4	CCDS47225.1	28	5	66459843	66459843	C	A	57	Missense	S1423R	B (0.302)
ALL-14	MUC17	CCDS34711.1	3	7	100676948	100676948	A	C	44	Missense	S751R	po D (0.618)
ALL-14	NPTX2	CCDS5657.1	4	7	98256583	98256583	T	C	47	Missense	L332P	pr D (0.998)
ALL-14	OR1D2	CCDS11019.1	1	17	2995888	2995888	C	T	48	Missense	A135T	B (0.008)
ALL-14	PCBP4	CCDS2839.1 CCDS2840.1	4,1	3	51994630	51994630	C	T	41	Missense	G56D,G22D	pr D (1)
ALL-14	PREX2	CCDS6201.1	23	8	69012074	69012074	A	T	21	Missense	K904I	po D (0.482)
ALL-14	SCG2	CCDS2457.1	1	2	224463709	224463709	G	A	48	Missense	H98Y	B (0.238)
ALL-14	SSH1	CCDS9121.1	12	12	109194580	109194580	G	A	21	Missense	A375V	po D (0.784)
ALL-14	TNRC6B	CCDS46713.1	5	22	40661049	40661049	A	G	42	Missense	N272S	B (0.009)

^In CCDS Reference Sequence

^^Numbering according to the Human Genome hg19 assembly

Abbreviations: T, tumor; N, normal; Ref nt, reference nucleotide; Var nt, variant nucleotide; AA, amino acid; fs, frameshift; D, deletion; nd, not determined (The PolyPhen-2 algorithm predicts only the impact of amino acid substitutions); B, benign; pr D, probably damaging; po D, possibly damaging.

Table S2. Enriched functional categories and pathways in discovery panel mutated genes (DAVID analysis).

	**Gene category**	**Enrichment score**
All cohorts	Extracellular structure organization	3.08*
	Plasma membrane	2.97*
	Neurological system process	1.87*
	Focal adhesion/ECM interaction	1.68*
	MHC protein complex	1.29*
	Small GTPase mediated signal transduction	1.28*
	Regulation of cell proliferation	0.99
	Cell migration	0.91
	Skeletal/Muscle development	0.87*
	Ion transport	0.81*
	Cell projection	0.78
	Sensory perception	0.74
	Protein kinase activity	0.72*
Children	Regulation of nervous system development	0.95
	Embryonic organ development	0.95*
	Cell projection organization	0.84*
	Ion transport	0.66
	Cell motion	0.61
	Regulation of cell proliferation	0.59*
	Transcriptional regulation activity	0.54
	Nucleotide binding	0.4
	G-protein coupled receptor activity	0.36
AYA	Growth factor binding	2*
	Plasma membrane part	2*
	Small GTPase mediated signal transduction	1.32*
	Extracellular matrix	1.1*
	Cell projection	1.09*
	Regulation of cell proliferation	1.05*
	Cell adhesion	0.85
	Protein kinase activity	0.82*
	Ion binding	0.57*
	Ion homeostasis	0.52
Adults	MHC/Cell adhesion	2*
	Regulation of apoptosis	0.77
	Neurological system process	0.71
	Plasma membrane	0.7
	Cell projection	0.65*
	Transcription regulation	0.21
	Nucleoplasm	0.16
	Ion binding	0.12

*Significant *p*-value.

Table S3. Validated mutations targeting recurrently mutated driver genes detected in the discovery and screening panel 1.

Gene	Total cases (81)	Children (15)	AYA (32)	Adults (34)
PAX5	12	0.00%	5 (15.60%)	7 (20.5%)
FLT3	9	1 (6.66%)	5 (15.60%)	3 (8.82%)
KRAS	9	3 (20%)	4 (12.5%)	2 (5.88%)
NRAS	7	3 (20%)	2 (6.25%)	2 (5.88%)
MLL2	5	1 (6.66%)	1 (3.12%)	3 (8.82%)
IL7R	4	0.00%	1 (3.12%)	4 (11.76%)
DNAH5	4	1 (6.66%)	2 (6.25%)	1 (2.94%)
ARID1A	4	0.00%	2 (6.25%)	2 (5.88%)
JAK2	4	0.00%	2 (6.25%)	4 (11.76%)
CREBBP	4	1 (6.66%)	1 (3.12%)	2 (5.88%)
ATM	2	0.00%	0.00%	2 (5.88%)
APC	2	1 (6.66%)	0.00%	1 (2.94%)
NOTCH1	2	1 (6.66%)	1 (3.12%)	0.00%
EP300	2	0.00%	2 (6.25%)	0.00%
TP53	2	0.00%	0.00%	2 (5.88%)
CDKN2A	2	0.00%	1 (3.12%)	1 (2.94%)
RUNX1	1	0.00%	1 (3.12%)	0.00%
JAK1	1	0.00%	0.00%	1 (2.94%)
NF1	1	0.00%	1 (3.12%)	0.00%

Table S4. Clinico-biologic features of the discovery panel.

Code	Age	Gender	Cohort	Molecular biology	Lekemic blasts (%)	Source of germline DNA	Protocol	Hb (g/dl)	Wbc (x10^9/L)	Plts (x10^9/L)
ALL_1	2	F	Children	Negative	89	CR sample	AIEOP LLA 2000	6.3	27.8	46
ALL_2	3	M	Children	Negative	97	CR sample	AIEOP LLA 2000	6.7	43.0	24
ALL_3	3	M	Children	Negative	83	Saliva	AIEOP LLA 2000	7.1	48.1	32
ALL_4	3.5	F	Children	Negative	90	CR sample	AIEOP LLA 2000	7.7	42.8	40
ALL_5	6	F	Children	Negative	87	Saliva	AIEOP LLA 2000	10.3	13.8	12
ALL_6	16	F	AYA	Negative	70	CR sample	LAL2000	8.5	2.8	95
ALL_7	16	F	AYA	Negative	90	CR sample	LAL0904	9.5	6.7	34
ALL_8	19	M	AYA	Negative	90	CR sample	LAL0904	10.5	109	9
ALL_9	24	F	AYA	Negative	73	CR sample	LAL0904	10.9	49	115
ALL_10	27	F	AYA	Negative	95	CR sample	LAL0904	8	8	10
ALL_11	47	F	Adult	Negative	90	CR sample	LAL0904	7.1	42	6
ALL_13	53	M	Adult	Negative	99	CR sample	LAL0904	8.1	22.2	18
ALL_14	57	M	Adult	Negative	80	CR sample	LAL0904	8.7	3.5	29

Abbreviations. CR, Complete remission.

Table S5. Characteristics of the B-NEG ALL patients included in screening panels 1 and 2.

	Cohort	Total N	Gender (M/F)	Hb average (g/dl)	WBC average ($x10^9/L$)	Plts average ($x10^9/L$)
**Screening panel 1**	Children	10	45/23	8.8 (4-14.3)	58.7 (1.28-357)	75.8 (5-394)
	AYA	27				
	Adults	31				
**Screening panel 2**	Children	35	54/34	16.7 (1.2-15.8)	25.7 (0.6-425)	62.4 (0.23-584)
	AYA	29				
	Adults	23				

Abbreviations. Hb, hemoglobin; WBC, white blood cell count; Plts, platelets count.

Table S6. List of adult and pediatric clinical trials.

Protocol ID	N of cases	NCT number	Protocol description
GIMEMA LAL2000	20	NCT00537550	Standard regimen for adult ALL
GIMEMA LAL0904	56	NCT00458848	Standard regimen for adult ALL
GIMEMA LAL1104	8	NCT00475280	Age-adjusted (elderly) standard therapy
GIMEMA LAL1308	11	NCT01156883	BFM-like therapeutic regimen
AIEOP LLA 2000	45	NCT00613457	BFM-based protoocol
AIEOP-BFM ALL 2009	10	NCT01117441	BFM-based protoocol

Table S7. Illumina sequencing summary.

Sample ID	Target region coverage (%)		Mean depth^	N of mapped reads (%)
	≥10X	≥30X		
ALL_1_T	87.09%	65.77%	56.42	98.88%
ALL_1_N	86.68%	63.23%	49.31	98.82%
ALL_2_T	87.89%	68.45%	57.95	98.78%
ALL_2_N	86.34%	62.33%	48.47	98.83%
ALL_3_T	86.85%	64.79%	52.97	98.86%
ALL_3_N	89.16%	72.97%	66.27	98.88%
ALL_4_T	84.09%	55.35%	43.24	98.88%
ALL_4_N	87.33%	69.46%	58.30	98.86%
ALL_5_T	88.80%	73.70%	69.37	98.85%
ALL_5_N	88.43%	69.24%	58.43	98.71%
ALL_6_T	89.31%	75.72%	72.16	98.88%
ALL_6_N	89.42%	76.31%	73.13	98.85%
ALL_7_T	87.14%	67.78%	56.47	98.79%
ALL_7_N	87.66%	68.67%	56.84	98.85%
ALL_8_T	89.10%	75.89%	74.60	98.74%
ALL_8_N	89.95%	78.34%	81.05	98.76%
ALL_9_T	85.14%	59.91%	45.74	98.79%
ALL_9_N	89.06%	73.65%	67.05	98.84%
ALL_10_T	87.40%	68.83%	57.67	98.73%
ALL_10_N	87.58%	68.92%	57.31	98.69%
ALL_11_T	87.01%	66.62%	54.53	98.68%
ALL_11_N	89.82%	79.14%	87.46	98.70%
ALL_13_T	88.07%	71.57%	62.89	98.78%
ALL_13_N	84.51%	55.30%	41.24	98.79%
ALL_14_T	88.55%	72.74%	66.04	98.76%
ALL_14_N	86.92%	66.75%	54.18	98.75%

^Mean number of sequence reads covering the target exome
Abbreviations: T, tumor DNA sample; N, germline DNA.

Table S8. Number of reads in CDS regions of selected driver genes in the screening panel 1.

Sample ID	*PAX5*	*FLT3*	*KRAS*	*NRAS*	*MLL2*	*IL7R*	*DNAH5*	*ARID1A*	*JAK2*
GQW-180	5902	2509	739	436	17033	1715	10827	11531	1108
GQW-181	11606	3025	751	860	19523	1862	11521	13447	2319
GQW-182	12370	3129	884	716	20044	2155	11510	14392	2637
GQW-184	8218	2293	702	630	15220	858	6610	11302	2240
GQW-185	9547	2834	689	714	17931	1765	10621	12396	2299
GQW-186	7697	2304	681	552	14065	1371	8509	10093	1794
GQW-187	7573	1010	332	620	13887	207	1993	7666	1175
GQW-188	13508	3482	1003	1015	20799	2180	14500	14951	3151
GQW-189	11348	2802	867	736	18213	1828	11491	13029	2301
GQW-190	9091	2388	712	677	14489	1629	10153	9973	2232
GQW-191	3654	1857	551	388	14114	1236	7253	9754	1600
GQW-192	13767	3428	839	822	23682	2116	12773	16122	2504
GQW-193	9458	2554	733	647	15805	1691	10113	11428	2132
GQW-194	11038	2883	890	792	20388	2051	11816	13757	2560
GQW-195	5382	4129	1066	1117	26935	2691	15790	18901	3393
GQW-196	8493	3922	1142	958	26336	2397	14373	17800	1459
GQW-197	15021	3634	894	861	23425	2336	13435	16926	2756
GQW-198	19840	1570	793	416	13130	1043	6737	10134	1278
GQW-199	7668	3342	676	868	21506	2167	12333	15708	1144
GQW-200	9997	3034	861	766	19884	1555	10351	13983	2253
GQW-202	17710	4293	1130	1153	30711	2885	17325	17551	3546
GQW-204	10629	1989	749	523	21658	1108	6800	13380	2324
GQW-205	18829	4231	759	1125	31546	2707	16334	21507	3094
GQW-206	11972	2753	754	708	26211	1516	10017	17506	2862
GQW-208	7601	2281	770	521	20695	1471	9226	13309	2249
GQW-209	9054	1876	526	509	17415	1356	8662	11313	1503
GQW-210	11176	2710	960	1254	41260	612	5091	23454	2485
GQW-211	9701	2451	715	652	14758	1653	9592	6109	2210
GQW-212	10307	3148	1218	712	19865	1667	11028	12853	3083
GQW-213	12789	1878	876	543	20104	837	6029	12156	2896
GQW-214	5377	3032	867	749	18194	1665	11831	12598	1243
GQW-215	7122	2254	1038	555	11780	1193	7150	9210	3078
GQW-216	23145	4741	1227	1392	47987	1668	13689	30452	3651
GQW-218	11865	3213	857	765	19131	1944	12105	13861	2425
GQW-219	4186	629	258	372	10792	272	1742	5322	873
GQW-221	5581	2697	701	685	14771	1740	10669	11236	1721
GQW-223	4909	2142	739	507	13050	1261	8468	9245	1907
GQW-224	4936	2883	945	768	16664	1619	11140	12115	2417
GQW-225	4798	2548	832	699	15047	1870	10437	10457	2368
GQW-226	6241	2293	615	444	14691	1449	8883	7960	1533
GQW-227	10606	2416	750	699	17418	1493	9820	12670	1883
GQW-228	11809	3021	812	808	21215	1875	11947	15267	2064
GQW-232	6442	1837	470	473	12402	1244	7276	8565	1488
GQW-233	13726	3575	1015	960	21326	2228	13734	15134	3041
GQW-234	10985	3033	1377	1333	29901	2840	18634	20984	2335
GQW-235	10897	2657	662	799	17462	1923	9944	12222	2003
GQW-236	12253	3281	959	862	23527	2018	13224	16096	2729
GQW-238	11509	3128	870	783	20066	1892	12311	13541	2419
GQW-239	8113	2206	611	540	14768	1373	8657	10633	1107
GQW-240	16535	4245	1160	1059	28982	2823	16718	19987	3222
GQW-241	12540	2879	671	752	20061	1821	11211	14166	2166
GQW-242	10194	3065	768	745	19571	1852	11360	13705	2368
GQW-246	12289	3339	952	836	20265	2010	12535	14266	2558
GQW-247	16432	4549	1299	1393	27991	2937	19148	20637	3873
GQW-248	5556	1842	486	470	11548	1130	7263	8300	1478
GQW-254	12944	3617	901	860	22813	2272	13906	16060	2706
GQW-255	6792	2097	592	578	14995	1439	8512	10305	1787
GQW-183	10110	3993	983	1049	25602	2453	14834	18900	2808
GQW-220	17048	2796	1613	1631	31592	1537	10979	16459	4699
GQW-222	10444	2587	638	678	18062	1701	10497	13058	2170
GQW-229	6219	2424	1347	440	16632	1285	6877	10008	4453
GQW-230	10899	2891	833	754	18674	1828	11092	13813	2275
GQW-231	8495	2635	1457	443	13842	1349	8516	11242	4575
GQW-237	10136	2286	794	610	16965	1378	8978	11620	2019
GQW-243	3864	2312	1029	400	12356	1110	6991	8728	3066
GQW-245	6977	2338	953	645	14844	1361	8323	10597	2853
GQW-249	7212	2202	672	541	14983	1230	6937	10543	2171
GQW-250	6343	1364	572	606	13929	1369	7952	9355	2056
CDS_length	10677	3006	1145	574	16668	1388	13954	13105	3422

Sample ID	*CREBBP*	*ATM*	*APC*	*NOTCH1*	*EP300*	*TP53*	*CDKN2A*	*RUNX1*	*JAK1*	*NF1*
GQW-180	10753	6256	6818	6760	9379	2119	111	1639	2719	17766
GQW-181	11808	6895	7275	5467	10465	2732	1683	2126	3112	13611
GQW-182	12418	7463	7726	7829	11652	3172	54	2261	3365	22090
GQW-184	5261	4728	2878	5697	8317	2354	2028	1629	2300	8499
GQW-185	11251	6442	7148	4974	9759	2412	1484	1905	2873	12478
GQW-186	8519	2938	6029	3839	7683	2082	140	1640	2221	10017
GQW-187	10580	1694	1001	6424	4679	1911	1805	1459	1136	4094
GQW-188	13030	9037	9490	5434	12043	3077	2021	2067	3663	16441
GQW-189	11162	6741	7534	4835	9713	2763	1780	1890	3080	13082
GQW-190	9282	9285	6414	3527	11960	2345	1363	1962	2645	12037
GQW-191	8381	4277	5415	3697	7294	1973	1225	1173	1870	9005
GQW-192	14075	7756	8815	6649	12986	3358	111	2271	3431	15420
GQW-193	9815	6105	6792	4474	8967	2259	1436	1384	2627	11228
GQW-194	11853	6728	8307	5311	10776	3197	1767	1963	2950	14076
GQW-195	16320	9869	10860	7151	14360	3792	38	2689	3918	19653
GQW-196	15364	8731	10558	7129	13932	3618	180	2699	3729	17510
GQW-197	14192	8214	8765	6201	12658	3352	1588	2150	3567	15997
GQW-198	7421	3930	3872	4733	6029	1585	172	1396	1531	8918
GQW-199	13053	6866	9014	5475	11831	3087	307	2990	3306	13038
GQW-200	12088	6779	6199	5267	10585	3480	1681	2006	2851	12626
GQW-202	18319	9804	11141	8364	15910	4399	2619	2801	4542	19691
GQW-204	12361	4640	4465	7324	8700	2469	1577	2515	1986	9187
GQW-205	18055	9719	10829	8981	16683	4487	67	2901	4566	19106
GQW-206	14338	5752	5670	8668	10682	3416	1960	3143	2866	12112
GQW-208	10339	5209	5890	5919	9113	2637	1607	2124	2466	10268
GQW-209	10625	3669	5481	6563	7007	3157	1664	4818	1742	11499
GQW-210	25369	4145	3988	13406	15276	5637	2602	4440	2743	10832
GQW-211	10003	5875	6338	4091	8598	2385	1542	1530	2669	11555
GQW-212	11273	7940	7117	4189	10620	4214	1122	2675	2614	22725
GQW-213	10013	3784	3612	7265	7544	2049	2297	3067	1902	9380
GQW-214	11380	7259	7700	4848	10721	2866	905	1793	2995	14374
GQW-215	6548	6951	4563	5035	5953	1705	1518	3495	1421	15902
GQW-216	29108	9285	7879	15285	21200	6698	541	3988	4290	19192
GQW-218	11797	7312	7682	5291	10862	2735	160	1991	3018	13976
GQW-219	6166	1437	1327	4159	2792	333	1245	1744	708	3417
GQW-221	10177	5926	7110	3704	9591	2367	1091	1265	2587	11802
GQW-223	8372	5269	6060	3161	7787	2018	1266	1265	2198	10150
GQW-224	10482	6985	7723	4436	9570	2540	63	1762	2882	13469
GQW-225	9986	6886	6900	3797	8824	2334	77	1806	2624	12249
GQW-226	6965	3981	6364	2915	8032	2353	813	1555	1582	10778
GQW-227	10226	5744	6341	4999	9566	2385	1819	1658	2523	11887
GQW-228	12599	6416	7604	6470	11244	2849	1908	1993	3283	13001
GQW-232	6992	14201	3007	3169	6586	1718	1142	1221	1870	9015
GQW-233	13374	8685	9418	5663	12179	3150	1947	2339	3548	16786
GQW-234	19156	11297	12290	5125	16477	4784	434	3192	4934	21878
GQW-235	10698	6412	6714	5094	9350	2880	1590	1790	2967	12110
GQW-236	13475	8203	9667	6193	11707	3118	1944	2434	3421	15970
GQW-238	11991	7485	8050	5048	10434	2777	1620	6287	3067	14663
GQW-239	8369	5446	6357	3875	8036	2168	246	1558	2127	10497
GQW-240	17388	10149	11483	7823	16414	3964	2428	2686	4164	19394
GQW-241	12352	6201	7353	5693	10705	3088	506	2263	2907	13374
GQW-242	11478	7141	8335	5054	10914	3132	1616	1578	2891	14118
GQW-246	12605	7558	8437	5455	11209	2938	1948	2130	3446	14364
GQW-247	17051	11946	12706	6487	16571	4348	2540	3189	4468	22527
GQW-248	6857	4435	5423	2922	6406	1749	1009	1106	1709	8408
GQW-254	14213	8061	9164	6302	12549	3356	2175	2374	3461	16019
GQW-255	8690	5253	6011	3776	7769	2054	233	1418	2243	10261
GQW-183	16073	8867	9706	7099	13968	3991	102	2726	3853	16479
GQW-220	20622	6578	5662	15687	9715	2440	6161	3637	2254	17696
GQW-222	10954	6598	6923	5161	9895	2610	1105	1793	2748	12452
GQW-229	6618	5486	4750	3238	6811	1276	776	1834	1367	12867
GQW-230	11937	6960	7153	5019	10019	3006	1640	2023	2912	13407
GQW-231	6140	6543	5091	4129	6455	1433	1203	1781	1392	13984
GQW-237	9913	5932	5427	4520	8849	2510	1467	1688	2437	11239
GQW-243	5810	5617	4636	2661	5561	941	325	1563	1441	11693
GQW-245	7914	5104	5656	3039	7788	1942	405	806	1990	12804
GQW-249	8336	4592	5231	3256	7076	1740	879	2391	2115	10807
GQW-250	8013	5571	5262	2851	7158	1102	595	1069	1955	11254
CDS_length	14605	9233	8547	7702	7276	3277	1382	3577	3489	18889

Table S9. Detail of primers including key (A) and MIDs (B) used to perform NGS of *JAK2*, *IL7R*, *CRLF2*, *PAX5*.

A

Primer name	Primer sequence
JAK2_E12_F	CGTATCGCCTCCCTCGCGCCATCAGACGAGTGCGTCCTCTTTGGAGCAATTCATAC
JAK2_E13_F	CGTATCGCCTCCCTCGCGCCATCAGACGAGTGCGTCGTTCTCCATCTTTACTCATTCTTT
JAK2_E14_F	CGTATCGCCTCCCTCGCGCCATCAGACGAGTGCGTTTATGGACAACAGTCAAACAACAA
JAK2_E15_F	CGTATCGCCTCCCTCGCGCCATCAGACGAGTGCGTAAAGTTGTGAGTTTTGCCAAT
JAK2_E16_F	CGTATCGCCTCCCTCGCGCCATCAGACGAGTGCGTCTCAATGCATGCCTCCAA
IL7R_E06_F	CGTATCGCCTCCCTCGCGCCATCAGACGAGTGCGTTGCATGGCTACTGAATGCTC
CRLF2_E06_F	CGTATCGCCTCCCTCGCGCCATCAGACGAGTGCGTCGCACGTCATGTTGAAAACT
PAX5_E02_F	CGTATCGCCTCCCTCGCGCCATCAGACGAGTGCGTGGGGCTCTGGGTCCTCAC
PAX5_E03_F	CGTATCGCCTCCCTCGCGCCATCAGACGAGTGCGTTTTCCTGGCCAGAGTAGCCCGT
JAK2_E12_R	CTATGCGCCTTGCCAGCCCGCTCAGACGAGTGCGTTTGCTAACATCTAACACAAGGT
JAK2_E13_R	CTATGCGCCTTGCCAGCCCGCTCAGACGAGTGCGTCCCACAAGAATGTATCCTCAGA
JAK2_E14_R	CTATGCGCCTTGCCAGCCCGCTCAGACGAGTGCGTTGTTTGGGCATTGTAACCTTC
JAK2_E15_R	CTATGCGCCTTGCCAGCCCGCTCAGACGAGTGCGTCACCTAACACAGACTATTTTACATG
JAK2_E16_R	CTATGCGCCTTGCCAGCCCGCTCAGACGAGTGCGTACAACATGCCCTTTACACC
IL7R_E06_R	CTATGCGCCTTGCCAGCCCGCTCAGACGAGTGCGTGGACAGCGTTTGCCTAATGT
CRLF2_E06_R	CTATGCGCCTTGCCAGCCCGCTCAGACGAGTGCGTCCATCATAAGAGTGGGCATTG
PAX5_E02_R	CTATGCGCCTTGCCAGCCCGCTCAGACGAGTGCGTTCAAGGGAAGCCTCGAGCT
PAX5_E03_R	CTATGCGCCTTGCCAGCCCGCTCAGACGAGTGCGTCCCAACCCCCACAGGCACGA

B

MID name	MID sequence
**MID 1**	ACGAGTGCGT
**MID 2**	ACGCTCGACA
**MID 3**	AGACGCACTC
**MID 4**	AGCACTGTAG
**MID 5**	ATCAGACACG
**MID 6**	ATATCGCGAG
**MID 8**	CTCGCGTGTC
**MID 10**	TCTCTATGCG

Figure S1. ***PAX5* mutations in B-NEG ALL. A)** Distribution and features of *PAX5* mutations. **B)** Incidence of *PAX5* mutations. **C)** Incidence of *PAX5* lesions.

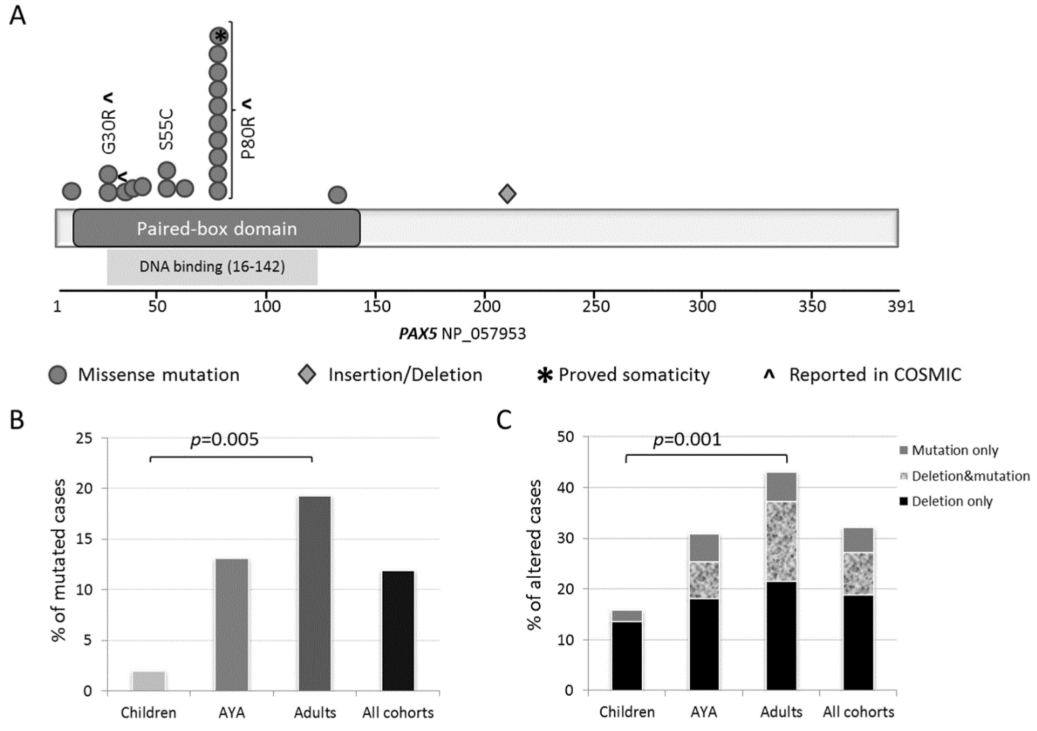

Figure S2. ***FLT3* mutations in B-NEG ALLs**. **A)** Type of *FLT3* mutations and frequency of *FLT3* mutations across age cohorts. **B)** Distribution and features of *FLT3* mutations.

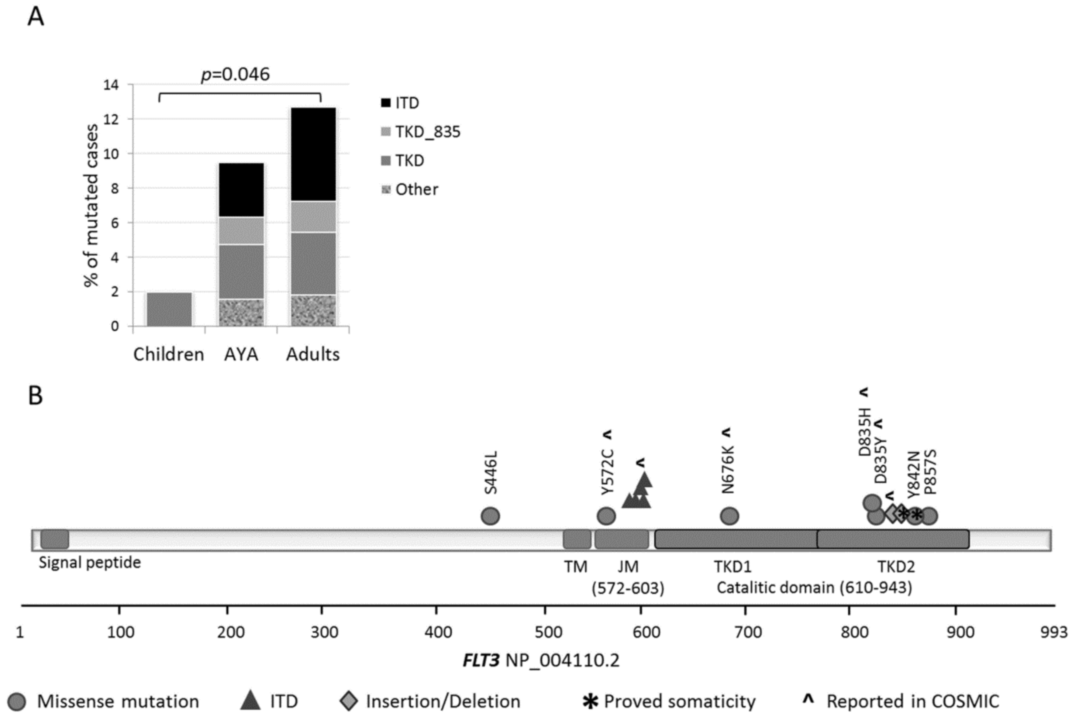

Figure S3. *KRAS* and *NRAS* mutations in B-NEG ALL. Distribution and features of *KRAS* **(A)** and *NRAS* **(B)** mutations.

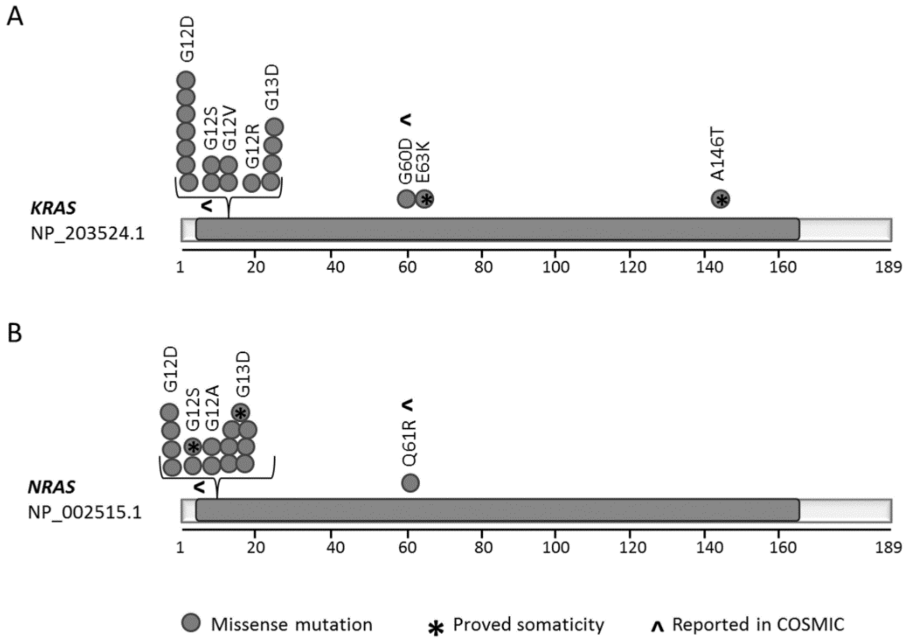

Figure S4. Heatmap of concomitant genetic features of the cases (N=21) harboring JAK/STAT pathway mutations.

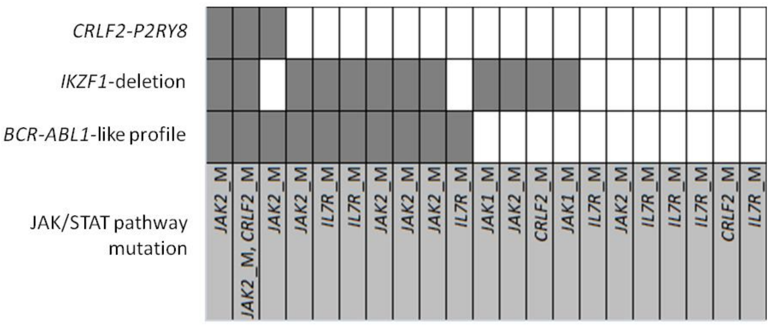

Figure S5. Experimental strategy.

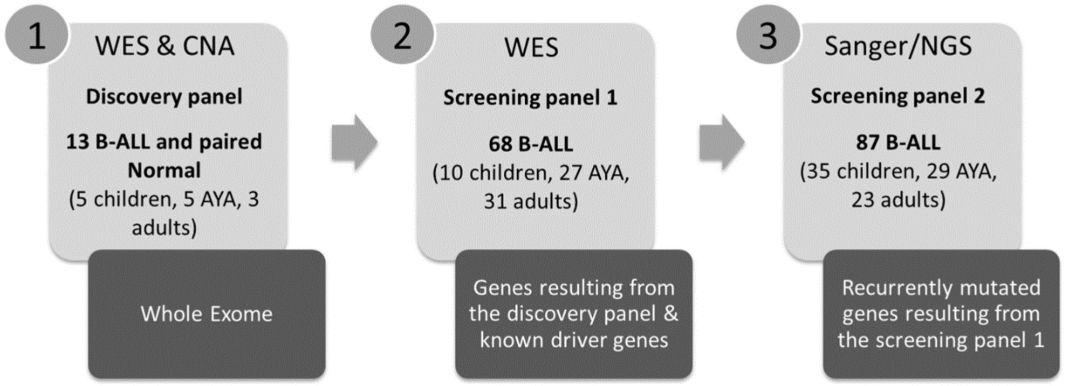

Supplement: Supplementary file 1 [file oncotarget-07-13886-s001.pdf]
